# A new class of protein sensor links spirochete pleomorphism, persistence, and chemotaxis

**DOI:** 10.1128/mbio.01598-23

**Published:** 2023-08-21

**Authors:** A. R. Muok, K. Kurniyati, C. K. Cassidy, F. A. Olsthoorn, D. R. Ortega, A. Sidi Mabrouk, C. Li, A. Briegel

**Affiliations:** 1 Institute of Biology, Leiden University, Leiden, The Netherlands; 2 Centre for Microbial Cell Biology, Leiden University, Leiden, The Netherlands; 3 Department of Oral and Craniofacial Molecular Biology, Philips Research Institute for Oral Health, Virginia Commonwealth University, Richmond, Virginia, USA; 4 Diamond Light Source, Harwell Science and Innovation Campus, Didcot, United Kingdom; The Ohio State University, Columbus, Ohio, USA

**Keywords:** chemotaxis, biofilms, protein sensor, cryoEM

## Abstract

Pathogenic spirochetes can alter their morphologies and behaviors to infect and survive within their hosts. Previous reports demonstrate that the formation of the so-called “round bodies” and biofilms, and chemotaxis are involved in spirochete pathogenesis. Here, we report a direct link between these cellular states that involve a new class of protein sensor with hitherto unclear function. Using cryo-electron microscopy, genetics, behavioral assays, and molecular modeling, we demonstrate that spirochetes regulate these behaviors in response to the small molecule *S*-adenosylmethionine (SAM) via a SAM sensor that is anchored to chemotaxis arrays. Furthermore, we establish an improved model for round body formation that now includes characterizations during log phase growth.

**IMPORTANCE:**

A new class of bacterial protein sensors monitors intracellular levels of *S*-adenosylmethionine to modulate cell morphology, chemotaxis, and biofilm formation. Simultaneous regulation of these behaviors enables bacterial pathogens to survive within their niche. This sensor, exemplified by *Treponema denticola* CheWS, is anchored to the chemotaxis array and its sensor domain is located below the chemotaxis rings. This position may allow the sensor to directly interact with the chemotaxis histidine kinase CheA. Collectively, these data establish a critical role of CheWS in pathogenesis and further illustrate the impact of studying non-canonical chemotaxis proteins.

## INTRODUCTION

Sensory systems allow microbes to adapt to and thrive in their environment and hosts. By sensing stimuli through ligand-specific proteins, bacteria can find nutrients and avoid toxic moieties, evade predators, adapt to their hosts or symbionts, and restructure their micro-environment. During these processes, bacteria often change their physiologies accordingly. For free-living bacteria, they allow the cells to find sources of food and withstand ecological changes (1). For bacterial pathogens, these systems can be critical for infecting hosts and sustaining infections (2, 3). Although numerous strides have been made in understanding how the underlying molecular networks that modulate these sensory systems operate, many key players and mechanisms remain unclear.

**Fig 1:**
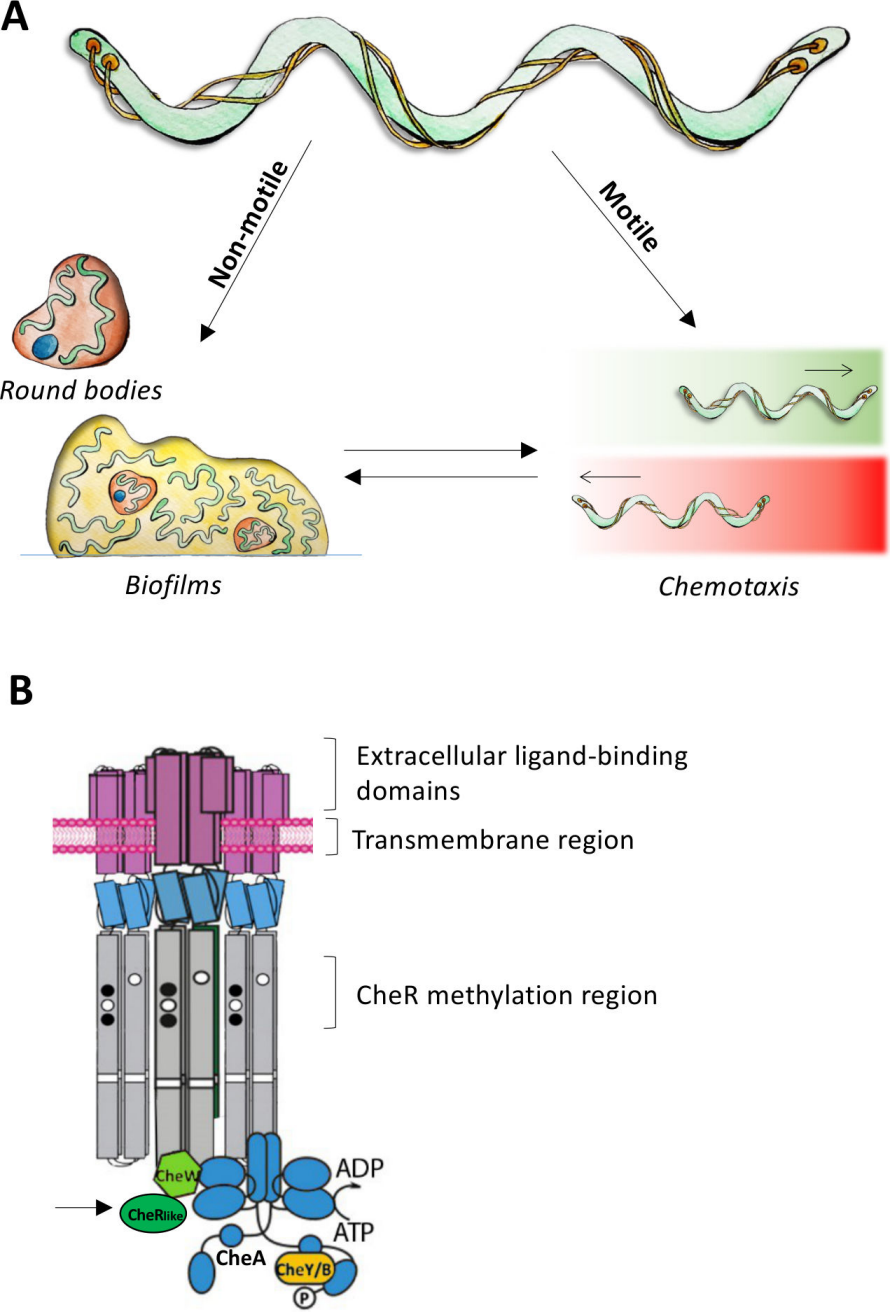
The life cycle and chemotaxis machinery of spirochetes. (**A**) Spirochetes can form nonmotile states that protect them from stress and contribute to host colonization. Motile spirochetes can undergo chemotaxis that enables them to move toward favorable environments (green gradient) and away from deleterious ones (red gradient). (**B**) The chemotaxis system consists of three main proteins: chemoreceptors that span the inner membrane, the kinase CheA (blue), and the adaptor protein CheW (green). *Td* CheW possesses an additional C-terminal domain, called the CheR_like_ domain (black arrow), that is a distant homolog of the CheR chemotaxis receptor methyltransferase.

For many bacteria, the appropriate response to some stimuli is to enter a non-motile cellular state. For example, when the environment is unfavorable (e.g., nutrient starvation), bacteria can form non-motile pleomorphic states and biofilms that protect the cells from external stresses (4 – 6). Pleomorphism describes the ability of microbes to alter their morphology in response to external conditions. When cells form biofilms, they cluster together and embed themselves in an extracellular matrix that offers physical protection from the environment. In pathogenic spirochetes such as *Treponema denticola* (*Td*)*,* a causative agent of periodontal diseases (2) and linked to Alzheimer’s disease (7, 8) and specific cancers (9), it is established that specific pleomorphic states and biofilm formation are involved in host colonization and infection persistence (Fig. 1A) (5, 10). These pleomorphic states are called “round bodies,” a form of non-motile cell with a minimized metabolic activity that is specific to spirochetes (10). These structures form as a response to stress to physically protect cells from the environment, and a single round body may contain several spirochete cells (4, 6, 11). These cells remain in a dormant state until the environment becomes favorable again, and the cells can resume normal growth and behaviors (12). Round body formation can be induced by a wide range of stresses including starvation (4), hypotonic treatment (13, 14), osmotic pressure (6), growth to stationary phase (15), and antibiotic exposure (15). In fact, round bodies can withstand otherwise lethal doses of some antibiotics (15, 16), which may account for the recurrence of spirochetal infections after antibiotic treatments (17). Round bodies are also the dominant cellular form in mature spirochete biofilms (17), spinal fluid (12), and brain tissues from Alzheimer’s patients (18). In addition to offering stress protection, spirochete biofilms are also formed in response to interactions with microbial symbionts and host molecules (19, 20).

It is unclear how chemical signals induce a switch from motile to non-motile forms. In other organisms, like *Comamonas testosteroni*, it is established that the chemotaxis system is directly involved in transitions from motile states to sessile biofilm states (21). Bacterial chemotaxis is the system that allows cells to sense their external environment through transmembrane chemoreceptors to ultimately control the motility apparatus of the cells, such as the flagella (22, 23). In general, the chemoreceptors recognize specific chemicals with their extracellular domains and transduce signals into the cell. There are also cytosolic chemoreceptors that recognize intracellular ligands. Two proteins, called CheA and CheW, bind to the intracellular tips of the receptors and initiate a phosphor-relay system when activated by the receptors (Fig. 1B) (24). CheA is a five-domain histidine kinase that directly activates its response regulators via phosphoryl-transfer (25). The protein CheW links CheA to receptors and collectively the three proteins form a membrane-associated hexagonal apparatus called the chemotaxis array. The initiated phosphor-relay subsequently controls the rotation direction of flagella, allowing the cell to move toward favorable environments and away from deleterious ones. It is established that chemotaxis is essential for the pathogenesis of some spirochetes. For example, *in vivo* experiments show that the chemotaxis system in *Borrelia burgdorferi* (*Bb*), the causative agent for Lyme disease, is essential for infecting mice (3, 26, 27).

In *Td*, its chemotaxis system is the only chemosensory system and possesses 20 chemoreceptors (28). Several chemotaxis proteins are essential for penetrating oral epithelial cells (2), forming monospecies biofilms (19), and forming synergistic polymicrobial biofilms (19, 20). Furthermore, it has been recently shown that *Td* cells can actively move from oral cavities to brain tissues where they induce structures associated with Alzheimer’s disease in mice (29), although the role of chemotaxis in this process is unknown. Recent research with *Td* has revealed that the structure of the chemotaxis system in spirochetes differs from canonical systems in several regards (30). Their chemotaxis arrays have unique linear symmetry and contain a novel CheW variant that possesses an additional *C*-terminal domain. Previous findings show that this additional domain, called CheR_like_, is widespread among the spirochaetes (Fig. 1B) (30, 31). It is a distant homolog of the chemotaxis protein CheR that methylates receptors using the methyl-donor *S*-adenosyl methionine (SAM) as part of an adaptation response (32, 33). In addition to its role in the chemotaxis system*,* SAM is a critical cofactor in methyl-transfer reactions that are essential for cell viability, both in eukaryotes and prokaryotes (34). In prokaryotes specifically, SAM is essential for the synthesis of some amino acids and may be important for microbiome interactions (35, 36). The CheR_like_ domain can bind SAM but does not possess the conserved residues for enzyme catalysis or receptor binding (30, 32, 37). Thus, the exact cellular role of this domain remained unclear.

Here, we applied cryo-electron tomography (cryo-ET) of *Td* cells to generate an improved model for spirochete round body formation. Namely, we show that log-phase *Td* cells form round bodies in a process similar to sporulation. This model suggests that spirochetes are capable of forming round bodies under non-stressed conditions, thus indicating higher virulence capabilities than previously thought. Furthermore, we show that the CheR_like_ domain influences round body and biofilm formation and functions as a chemotaxis sensor for SAM. This finding, exemplified in *Td*, links transition from motile chemotactic cells to non-motile round bodies and biofilms. This is the first reported instance of a characterized CheW-fused chemotaxis sensor, which behests a new designation for this protein, which we have called CheWS (CheW SAM-sensor).

## RESULTS

### Round body formation in *T. denticola*

Spirochetes are typically present as long, thin spiral-shaped cells that are motile and chemotactic but can form round bodies and biofilms. Round bodies have been investigated in the spirochetes *Td* (38)*, Bb* (4, 5, 39), and *Treponema pallidum (Tp*) (40) through microscopy methods and have led to an established model for their formation (Fig. S1) (10). This process generally occurs in two steps. First, the spiral-shaped cell significantly enlarges its outer membrane at the cell tip so that the cell can then move into that newly formed space. Once the cell is inside this protective membrane, it can divide to form typical spiral-shaped cells. Additionally, the round bodies may also contain dense, spherical cells called “core structures” that are typically dominant in aged round bodies (13). Here, the encased bacteria can “hide” from the unfavorable environment and avoid cell death. When these round bodies are placed back into favorable, non-stressful environments, they form motile cells within a few days (5). However, the core structures may take several weeks before forming the spiral-shaped, motile cells (5, 12, 13). The ability of spirochetes to form round bodies has been described as a form of pleomorphism, as the cells are changing their morphology and metabolism in response to their environment.

**Fig 2:**
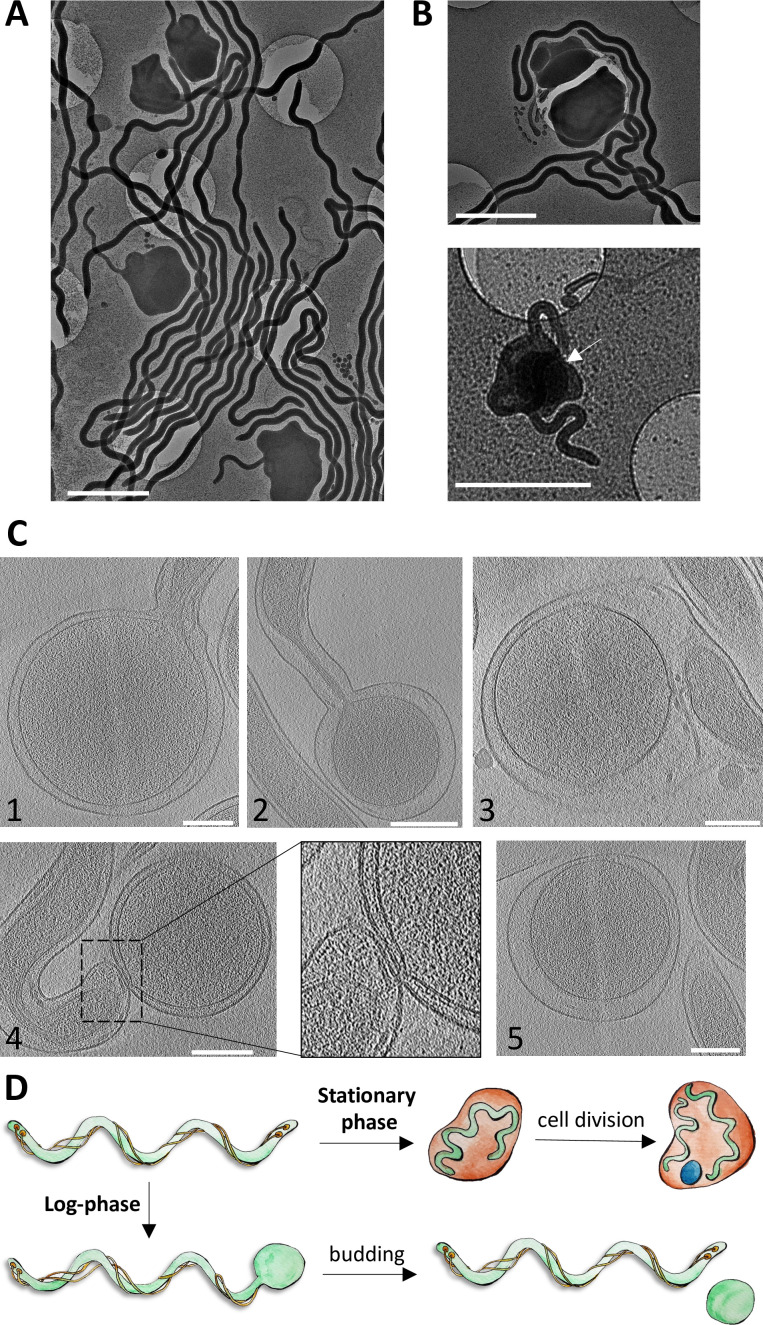
Cryo-electron microscopy reveals the morphology of *Td* round bodies. (**A**) In the stationary phase, *Td* forms round bodies that resemble morphologies seen in previous reports. Scale bars are 2 µm. (**B**) The stationary phase round bodies may contain more than one spiral-shaped spirochete (top), or “core structure” (white arrow) (bottom). Scale bars are 2 µm. (**C**) During log-phase, round bodies do not contain spiral-shaped cells, are smaller than stationary phase round bodies, and bud from the tips of spiral-shaped cells. The formation of these round bodies is shown in steps 1–5. 1: The outer and inner membranes of the cell tips begin to expand. 2: The inner membrane begins to pinch off from the forming round body. 3: The inner membrane of the round body and spiral cell is no longer connected but they are still attached via the outer membrane. 4: The outer membrane of the round body starts to disconnect from the spiral cell. 5: The round body is now separated from the spiral cell. Scale bars are 0.2 µm. (**D**) Log phase round body characterizations generate an improved model for round body formation, which previously only included stationary phase characterizations.

These previous investigations of the round body formation were limited to traditional electron microscopy (EM) and light microscopy methods. As such, structural insights into this process have been lacking. Therefore, we conducted cryo-EM and cryo-ET experiments with *Td* wild-type (WT) cells undergoing round body formation. We observed notable differences between cells in the log phase and stationary phase growth. During the stationary phase, our images match the established model for round body formation (Fig. 2A). Large round bodies (~1–2 µm) (38) are present and the typical spiral-shaped spirochete cells are clearly visible inside the round bodies (Fig. 2A and B). Several of these round bodies contain more than one cell, including core structures observed in other spirochetes (Fig. 2B) (12, 13). However, during the log phase, we see much smaller (200–400 nm), densely packed round bodies that form at the cell tips (Fig. 2C). These round bodies ultimately pinch off from the mother cell. In the early stages of this process, both the outer and inner membranes of the mother cell and round body are connected. At later stages, only the outer membrane is connected, which then separates to form a free round body and an intact mother cell (Fig. 2C). Unlike the established model, we neither see enlarged vacant outer membranes nor spiral cells inside the round body at any stage. Instead, the round bodies resemble the previously described electron-dense core structures (12, 13). Therefore, in the log phase growth, round body formation has a stronger resemblance to a sporulation process rather than pleomorphism. Collectively, these results were used to generate a more comprehensive model for round body formation that now includes log phase characterizations (Fig. 2D).

The log phase round bodies possess two membranes and a continuous peptidoglycan layer during all stages of growth and after separation from the mother cell (Fig. S2). Additionally, some round bodies possess flagella and flagellar motors between the outer and inner membranes (Fig. S3). The round bodies do not contain visible chemoreceptor arrays. However, in some instances, arrays are visible within the mother cell in regions adjacent to the forming round body (Fig. S3).

### Interactions of the CheWS protein

The *Td* CheWS protein has been demonstrated to interact with *Td* CheA and bind SAM with a Kd of ~9 µM (30). Isothermal calorimetry experiments of purified CheWS show that the protein binds *S*-adenosyl homocysteine (SAH) with a Kd of ~17 µM (Table S1; Fig. S4). The isolated CheR_like_ domain of CheWS also binds SAM and SAH with a Kd of ~18 µM and ~35 µM, respectively (Table S1; Fig. S4). A CheR_like_ domain mutant that is mutated in the hypothetical SAM-binding pocket (E297A, D321A) does not produce a measurable interaction with either SAM or SAH (Table S1; Fig. S4). These results indicate that the CheW domain of CheWS contributes to the observed affinity for both SAM and SAH, perhaps through stabilization of the CheR_like_ domain. Additionally, CheWS binds SAM with higher affinity than SAH, indicating that SAM is the preferential substrate.

### The CheR_like_ domain of CheWS influences round body formation

**Fig 3:**
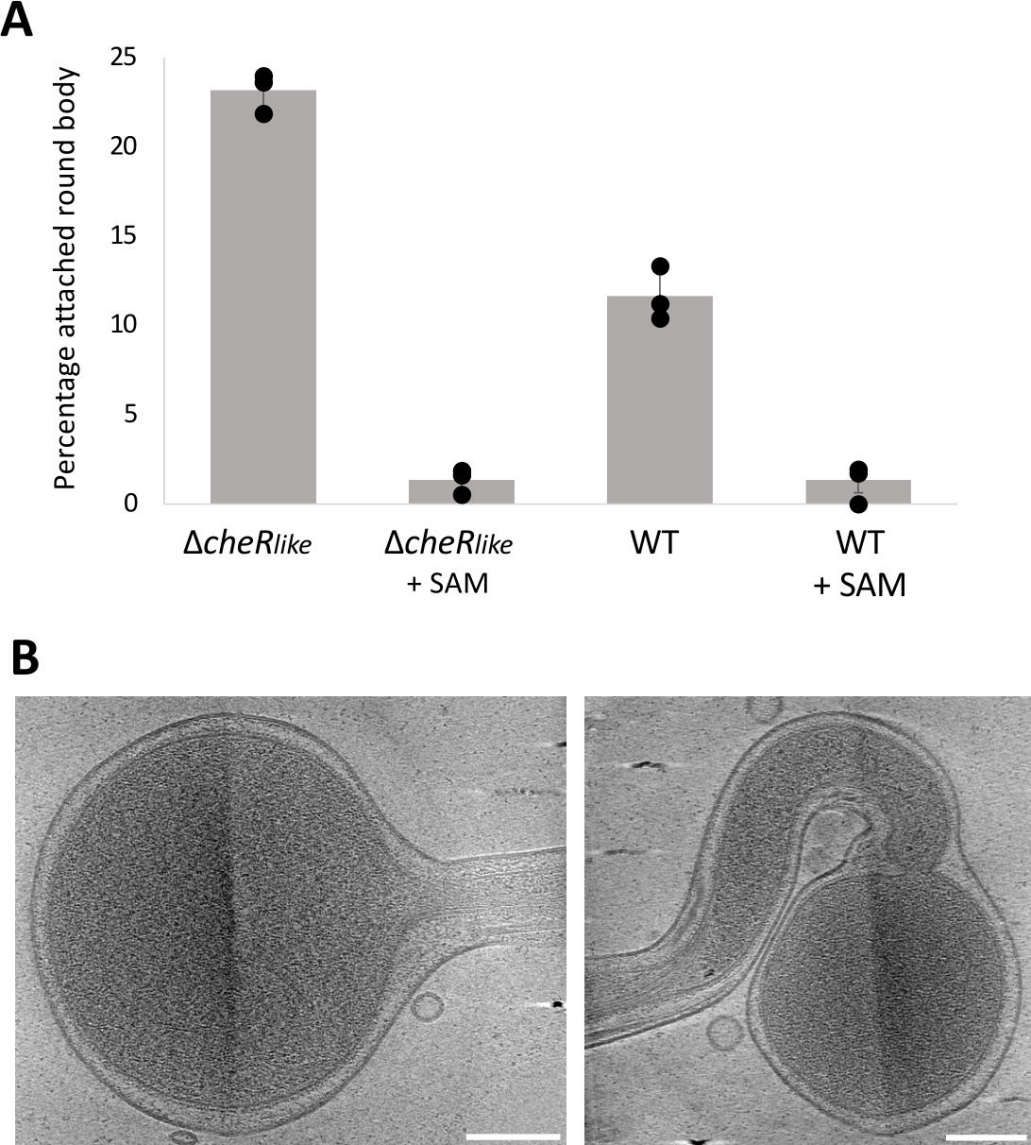
*Td* round body formation is influenced by the CheR_like_ domain. (**A**) The percentage of cells undergoing log phase round body formation was quantified in *Td* WT and a *Td*ΔCheR_like_ strain. The WT and ΔCheR_like_ strains have ~11.6% ± 1.51% and ~23.2% ± 1.1% of cells undergoing round body formation, respectively. There is a significant difference between the two strains using a two-tailed null hypothesis significance test (*P* < 0.05). When 0.5 mM SAM is added to the cells, the WT and ΔCheR_like_ strains have ~1.2% ± 1.1% and ~1.4% ± 0.7% of cells undergoing round body formation, respectively, and there is no significant difference between the two strains (*P* > 0.05). However, for both strains, the presence of SAM significantly reduces the percentage of cells undergoing round body formation (*P* < 0.05). Results are expressed as the mean of cell percentages undergoing round body formation for three samples ± 1 SD. (**B**) Log phase round bodies in the ΔCheR_like_ strain are morphologically similar to the WT strain. Scale bars are 0.2 µm.

Previous cryo-ET experiments of motile (spiral-shaped) WT and Δ*cheR_like_* cells have revealed a significant difference in the structural stabilization of chemotaxis arrays between the two strains (30). Here, through cryo-EM, we also observed a significant difference in the fraction of log phase cells that are in the process of forming round bodies. For the WT and Δ*cheR_like_* strains, ~11.6% and ~23.2% of the cells are observed forming round bodies, respectively (Fig. 3A). The addition of 0.5 mM SAM almost completely eliminates observed round body formation in both strains, with no significant difference found between them (Fig. 3A). These results are summarized and compared in Fig. S5. These data show no discernible phenotypic difference between the two strains (Fig. 3B).

### The CheR_like_ domain of CheWS influences biofilm formation

**Fig 4:**
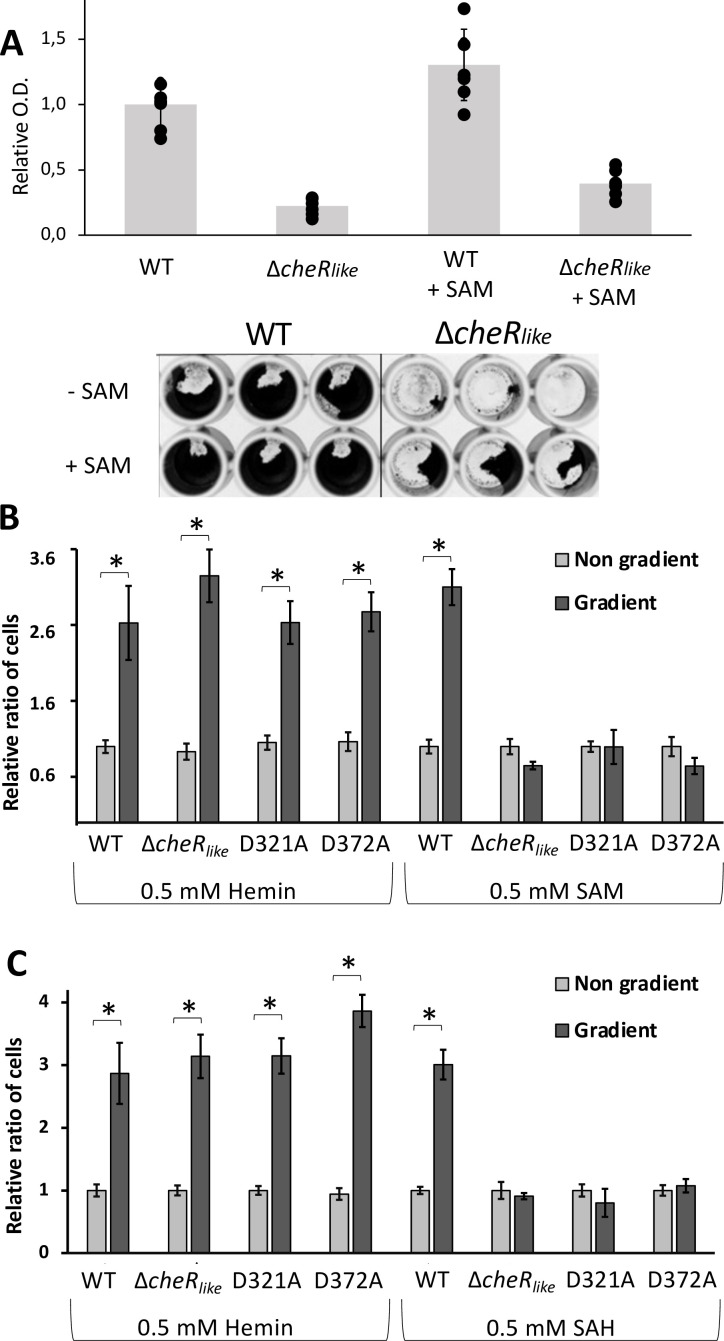
The CheR_like_ domain influences biofilm formation and chemotaxis toward SAM. (**A**) Quantitative biofilm assays show that the CheR_like_ domain significantly alters biofilm formation. The relative ratio of biofilms formed for the WT and Δ*cheR_like_* strains are 1.0 ± 0.17 and 0.22 ± 0.06, respectively. There is a significant difference between the two strains using a two-tailed null hypothesis significance test (*P* < 0.05). When 0.5 mM SAM is added to the samples, the amount of relative biofilm for the WT and Δ*cheR_like_* strains are 1.30 ± 0.27 and 0.39 ± 0.10. There is a significant difference in biofilm formation between the two strains in the presence of SAM (*P* < 0.05). Furthermore, for both strains, the addition of SAM significantly increases the relative amount of biofilm (*P* < 0.05). Results are normalized so that the mean for the WT strain without SAM added is equal to 1. Results are expressed as the mean OD_595_ for seven samples ± 1 SD. The bottom image is a representative experiment for relative biofilm quantification. (**B**) SAM chemotaxis assay of *T. denticola* wild-type (WT), Δ*cheRlike*, D321A, and D372A strains using hemin as a positive control. For the non-gradient control, both the capillary tubes and bacterial suspensions contained the same concentration of tested attractants. The results are represented as the mean of cell numbers ± SEM. The data were statistically analyzed by one-way analysis of variance followed by Tukey’s multiple comparisons at *P* < 0.01. The data are normalized so that the control samples are equal to 1. (**C**) Identical SAH experiments were conducted as in (**B**).

As the Δ*cheR_like_* strain has a significant increase in cells undergoing round body formation, we conducted assays to determine the subsequent impact on biofilm formation. Quantitative absorbance-based biofilm assays with the WT and *ΔcheR_like_* strains reveal that the *ΔcheR_like_* strain produces significantly less biofilm than the WT strain (Fig. 4A). The addition of 0.5 mM SAM significantly increases biofilm formation in both strains, but the *ΔcheR_like_* strain still forms significantly less biofilms than WT (Fig. 4A). Planktonic growth assays demonstrate that the addition of SAM does not alter stationary phase growth (Fig. S6A). These results are summarized and compared in Fig. S5.

### The CheR_like_ domain of CheWS modulates chemoattraction toward SAM and SAH

Previous research shows that the CheR_like_ domain does not possess the conserved residues for catalysis or receptor binding, but that it does bind SAM (30). Therefore, we conducted capillary-based chemotaxis assays to determine if the CheR_like_ domain functions as a sensor for SAM and/or SAH. While it was previously unknown if *Td* chemotactically responds to SAM and/or SAH, capillary assays with the WT strain demonstrate that they are both a chemoattractant. However, the *ΔcheR_like_* strain does not respond to either attractant and the response is not significantly different from non-gradient negative controls (Fig. 4B and C). Additionally, strains that are mutated at a single residue in the SAM/SAH-binding pocket of the CheR_like_ domain (D321A and D372A) do not respond to either ligand (Fig. 4B and C; Fig. S6B, Table 1). As a positive control, chemoattraction toward the established attractant hemin was also tested (41). Both the WT and *ΔcheR_like_* strains are attracted to hemin, demonstrating that chemotaxis to known attractant is not impaired (Fig. 4B and C). These results are summarized and compared in Fig. S5. Taken together, these results support that the CheR_like_ domain specifically functions as a sensor of SAM and SAH.

### Structural analysis of *Td* CheWS

Crystallography experiments of the CheWS protein were previously attempted but unsuccessful (30). Crystallography experiments of the isolated CheR_like_ domain (residues 203–444) were also unsuccessful, with no visible crystals forming in any tested conditions. When the CheR_like_ domain is expressed and purified without the *N*-terminal sub-domain (residues 258–444), the protein precipitates at ambient temperatures, indicating that smaller *N*-terminal sub-domain is critical for the stability of the *C*-terminal sub-domain.

**Fig 5:**
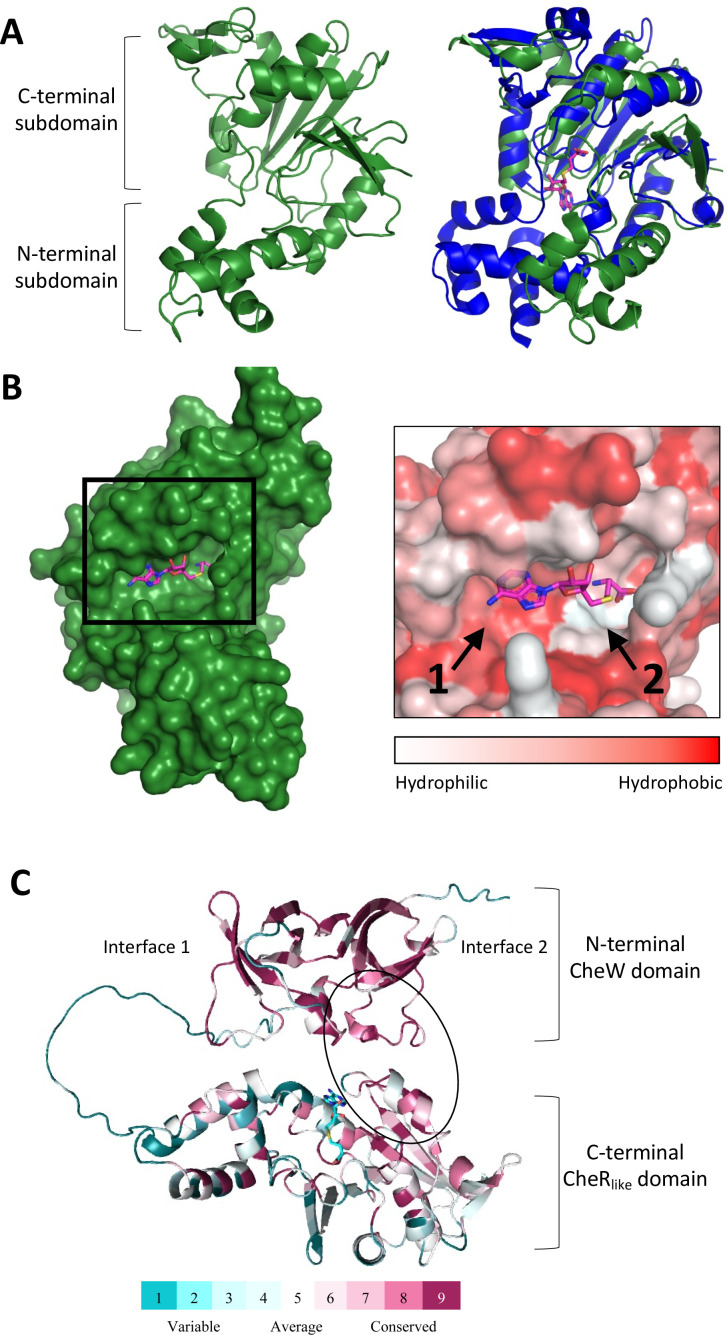
Structural modeling and conservation analysis of the *Td* CheR_like_ domain. (**A**) The CheR_like_ domain model (green) possesses the *N*-terminal and *C*-terminal sub-domains of classical CheR proteins (left). However, when compared to known structures of classical CheR proteins (blue, PDBID: 5FTW), the relative position of the *N*-terminal sub-domain is shifted (right). (**B**) These observed structural differences in the CheR_like_ domain model expose the hypothetical SAM pocket to the solution (left). The hypothetical SAM pocket is relatively hydrophobic near the adenine ring of SAH (arrow 1) but relatively polar near the homocysteine portion (arrow 2) (right). (**C**) Conservation analysis of the CheWS model shows that the *N*-terminal CheW domain and the *C*-terminal CheR_like_ domain interact at a highly conserved region above the SAM-binding pocket (black circle). The interaction site of the two domains does not overlap with interface 1 and interface 2 of CheW. SAM is shown as a stick model (teal).

A model of the *Td* CheR_like_ domain from the CheWS protein (residues 203–444) was determined using AlphaFold2 (42). The software produced five models that slightly vary in the Local Difference Distance Test and PAE scores (Fig. S7). We further analyzed the highest scored model (model 1). Like classical CheR proteins, the CheR_like_ model is a two-domain, mixed α/β protein (Fig. 5A) (32, 43). The *N*-terminal domain consists of three helices and is much smaller than the mixed *C*-terminal domain. The *C*-terminal domain possesses an obvious cavity located at the expected SAM-binding region (Fig. 5B). We also generated an AlphaFold model of the classical CheR protein in *Td*, which also does not have an experimentally derived structure (Fig. S8A).

While the CheR_like_ domain possesses the typical topology of classical CheR proteins, there are differences in tertiary interactions that result in a relative displacement of the *N*-terminal sub-domain (Fig. 5A). Specifically, in the CheR_like_ domain, several loops of the *N*-terminal sub-domain interact with loops of the central β-sheet in the *C*-terminal domain through hydrophobic contacts. The relative position of the *N*-terminal sub-domain of the CheR_like_ domain exposes the expected SAM-binding pocket to the solution (Fig. 5A and B). In known classical CheR structures, and our *Td* classical CheR model, the substrate is buried in a cavity that is produced by interactions between the *N*- and *C*-terminal sub-domains (Fig. 5A; Fig. S8A) (32). When the closest-related classical CheR homolog of known structure (*Bacillus subtilis* CheR with the SAM analog, SAH, PDBID: 5FTW) (43) is aligned to the CheR_like_ structure, the SAH molecule in 5FTW fits perfectly within the expected substrate pocket of the CheR_like_ domain (Fig. 5B). Residue mapping using the Eiseberg hydrophobicity scale (PyMol) reveals that the CheR_like_ substrate pocket has high hydrophobicity near the adenine ring but is highly polar near the homocysteine portion of the molecule, which is also seen in known classical CheR structures (Fig. 5B) (32, 37, 43).

To investigate potential interaction regions of the CheR_like_ domain, the residue sequence conservation using spirochete CheR_like_ domains was mapped onto the AlphaFold structure (using ConSurf with prealigned input sequences). The sequences used for this analysis were all the previously identified CheR_like_ domains that are exclusive to spirochetes (30). Overall, the CheR_like_ domain has high sequence conservation on two adjacent surface-exposed patches, which are both located on the *C*-terminal subdomain (Fig. S8B). One conserved patch corresponds to the substrate-binding pocket, where the residues W256, G291, C292, E297, D321, H371, R388, and D389 are strictly conserved. The second patch is located on a “lid” directly above the substrate pocket, where residues D323, L324, S328, F368, E369, Y370, H371, and D372 are strictly conserved (Fig. S8B). In the *N*-terminal subdomain, there are five strictly conserved residues that are at least partially surface-exposed, but they do not form an obvious patch, and most of them are located at the interface with the *C*-terminal subdomain. In total, ~14.5% of residues in the CheR_like_ domain are strictly conserved (35/241 residues). Identical analyses were conducted using the *Td* classical CheR AlphaFold model and previously identified F2 spirochete CheR sequences (30). In the classical CheR proteins, ~25.6% of all residues are strictly conserved (69/269 residues). Conserved surface-exposed patches are located on areas that surround the buried substrate pocket, the *N*-terminal subdomain, and the *C*-terminal region known to interact with receptor substrates (Fig. S8C) (37). Notably, the classical CheR does not possess the conserved, surface-exposed “lid” above the substrate pocket since the ligand is buried (Fig. S8C). Additionally, the CheR_like_ domain is not conserved at the receptor-binding regions of classical CheRs (Fig. S8B and C) (37).

To determine if the CheR_like_ domain interacts with the CheW domain of CheWS, the full-length protein was modeled using AlphaFold. The highest ranked model shows that the CheR_like_ domain directly interacts with the CheW domain (Fig. 5C). This interaction does not occur on the CheW interfaces that are known to interact with the CheA P5 domain, called Interface one and Interface 2, and does not preclude the CheR_like_ substrate pocket (Fig. 5C) (25). Sequence conservation analysis of the model shows that the highly conserved CheR_like_ “lid” interacts with a conserved patch on the CheW domain (Fig. 5C; Fig. S9A).

### Structural analyses of *Td* chemotaxis rings

**Fig 6:**
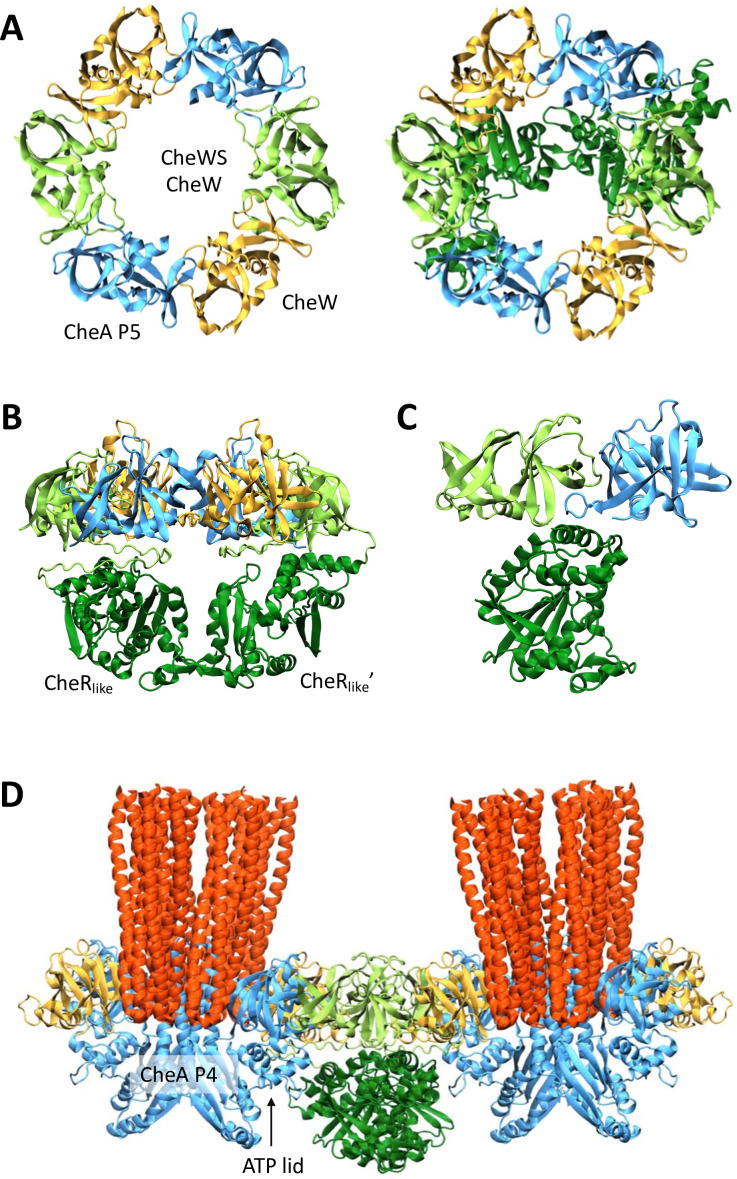
Structural modeling of the Td rings. (**A**) AlphaFold model of a Td ring without the CheR_like_ domains (left) and with the CheR_like_ domains included (right). CheA P5 is shown in blue, CheW in gold, the CheW domain of CheWS in light green, and the CheR_like_ domain in dark green. (**B**) Model of Td ring including the CheR_like_ domains shown from the side. (**C**) Zoom into the binding mode between a CheR_like_ domain and the hexameric portion of the td ring. (**D**) Alignment of the chemotaxis ternary complex (PDB 6SIK) with the *Td* ring model places the CheR_like_ domains next to the CheA P4 catalytic domains. Chemoreceptors are shown in red.

In chemotaxis arrays, CheA and CheW interact to form six-subunit rings. In canonical chemotaxis systems, these rings are composed of alternating CheA P5 domains and a CheW protein (22). In the *Td* system, these rings consist of CheA P5 and two CheW proteins (a classical CheW protein and CheWS), which allows CheA to have a strict linear arrangement within the arrays (30). To investigate the structure of the *Td* chemotaxis ring, we used AlphaFold to generate models of two copies each of *Td* CheA P5, the classical CheW protein, and CheWS with and without the CheR_like_ domain. In both cases, the highest ranked model showed a hexameric ring with the expected domain distribution (Fig. 6A). Intriguingly, in the model containing full-length CheWS, the CheR_like_ domains were positioned below the ring structure, spanning the central cavity (Fig. 6A through C). This arrangement is consistent with previous hypotheses based on cryo-ET experiments demonstrating an unknown density in the center of *Td* rings (30). Comparison of the CheWS structure observed in the ring model with the AlphaFold model of isolated full-length CheWS shows that the relative orientation between the CheW and CheR_like_ domains varies considerably. Overlaying the CheW domains of the two models places the CheR_like_ domain from isolated CheWS directly in the center of the ring, creating severe clashes with the remaining ring structure. Such a rearrangement may therefore be necessary to accommodate the formation of hexameric rings and extended arrays.

The two CheR_like_ domains were further observed to have slightly different positions relative to the ring structure. Each domain, nevertheless, directly interacts with its associated CheW domain via helices on the *N*-terminal and *C*-terminal subdomains (Fig. 6B and C). Additionally, the CheR_like_ domains interact with the neighboring CheA P5 domain via the *C*-terminal subdomain (Fig. 6C), while weaker contacts were observed between the *N*-terminal subdomains of the two CheR_like_ domains (Fig. 6A and B). Notably, the relative orientation of these domains leaves the highly conserved substrate-binding pocket and “lid” regions on the CheR_like_ domains solvent exposed. Comparison of the *Td* ring models with and without the CheR_like_ domain shows that the conformation of the ring is virtually unaltered. It is possible therefore that these additional inter-domain contacts may affect the overall stability and/or flexibility of the *Td* ring structure, perhaps to enable extended arrays to accommodate the high membrane curvature of *Td* cells (30).

To assess the positioning of the CheR_like_ domain in the context of the receptors and CheA, we further aligned a model of the ternary chemotaxis complex (PDB 6SIK) (44) to the *Td* ring model. The resulting overlay produced no contacts between the CheR_like_ domain and receptors. Rather, the *N*-terminal subdomain of the CheR_like_ domain was observed to lie directly adjacent to the CheA P4 catalytic domain and ATP lid (Fig. 6D; Fig. S9B). It is therefore conceivable that the CheR_like_ domain could modulate CheA activity through SAM-mediated conformational changes that affect ATP binding and/or CheA P4 dynamics (45, 46).

### The CheW protein domain is present in a diverse set of proteins

Given the role of CheWS in signal input, we investigated if the CheW protein domain is also present in other proteins besides the known CheV (47), CheA (22), 2xCheW (28), 3xCheW (48), and CheW-CZB (49). Using the Pfam database, we found 30 unique domain architectures that contain the CheW protein domain model that have not been described in the literature to date (Fig. S10). From this data set, 12 were associated with some other domain proteins related to signal input according to the domain classification from the MiST3 database (50).

## DISCUSSION

Here, we revised the model for round body formation in spirochetes and established a link between round body formation and chemotaxis. An explanation for our observed deviation from the established model for round body formation could be that the previous visual characterizations utilized spirochete cells under environmental stress [reviewed in Bamm et al. (10)], which increases round body prevalence. Additionally, these studies used traditional transmission or scanning electron microscopy techniques that require cell dehydration and/or chemical processing (51). Cryo-ET, on the other hand, instantaneously freezes cells in vitreous ice without chemical treatment, reducing artifacts that result from cellular stress or chemical treatments (51). Our results support the previous observation during stationary phase growth, where nutrients are depleted and/or growth inhibitory chemicals are accumulated. However, in log phase growth, where the cells are under more ideal metabolic conditions, we observed a not-yet described round body formation process. Under these conditions, round bodies are phenotypically distinctive and seemingly formed by a different mechanism. Log phase round bodies morphologically differ from established stress-induced round bodies in three aspects: they are significantly smaller, have a singular dense structure in the inner membrane, and do not contain spiral-shaped cells. These round bodies form by budding and pinching off from the cell, as opposed to forming a vacant outer membrane compartment seen during the stationary phase (5, 39). Therefore, we propose that spirochetes in favorable conditions undergo a sporulation-like process to produce round bodies that resemble previously characterized “core structures” (12, 13). This process could be advantageous to spirochetes since core structures may stay dormant for several weeks (5, 13), thereby ensuring colony occupancy if conditions become suddenly unfavorable. If this hypothesis is true, pathogenic spirochetes are more virulent and persistent than originally thought—the cells are not just capable of protecting themselves from deleterious environments but they also continuously shed germination-delayed round bodies as a fail-safe for colony collapse. Notably, a previous study in *Bb* investigated the presence of round bodies in log phase and stationary phase cultures through traditional fluorescence microscopy (15). This study reported that round bodies are not present during the log phase. However, the images contain small spherical puncta that may be round bodies, but their small size make them difficult to convincingly detect in these resolution-limited light microscopy images.

Spirochete growth and peptidoglycan (PG) synthesis occur at specific mid-cell sites where binary fission eventually ensues (52). In contrast, the cell poles are regions where no cell wall expansion occurs (52). Therefore, non-specific or uncontrolled blebbing at the cell tips due to PG growth is unlikely a determining factor for round body formation. In support of this, our cryo-ET experiments clearly demonstrate the presence of a continuous PG layer around both the forming and disassociated log phase round bodies. As the log phase round bodies possess the PG layer and two membranes, they do not resemble outer-membrane vesicles that have been previously observed in spirochetes (53).

We found that a specific protein that is associated with the bacterial chemotaxis system, called CheWS, serves as a functional link between chemotaxis and round body formation. Specifically, CheWS possesses a *C*-terminal CheR_like_ domain that functions as a SAM sensor to modulate these behaviors. In the *Td* WT strain, the presence of SAM decreases round body formation and increases biofilm formation. An exact opposite trend is produced when the CheR_like_ domain is absent in *Td*; round body formation increases and biofilm formation decreases. Collectively, these data indicate that the CheR_like_ domain is involved in physiological transitions that are modulated by SAM. Notably, samples that contain the WT strain without the addition of SAM do not produce identical results to the Δ*cheR_like_* strain without SAM. These effects may be due to the fact the WT strain can still sense and respond to intracellular SAM levels that are sustained without exogenous SAM addition. However, the interpretation of the Δ*cheR_like_* experiments with SAM is less clear. Round body and biofilm quantification in the Δ*cheR_like_* strain differs significantly when exogenous SAM is present. Therefore, the role of CheR_like_ in round body/biofilm formation could be more complex and involve additional interaction partners, or a separate system can still sense and respond to exogenous SAM. Nevertheless, it is clear that states linked to spirochete persistence are influenced by SAM and the CheR_like_ domain. A previous study shows that when *Td* is incubated with other oral pathogens, co-biofilms are more abundant, and round body formation is reduced when compared to monospecies *Td* biofilms (54). As the addition of exogenous SAM produces similar effects, it is possible that small molecules such as SAM may act as chemical messengers to facilitate these transitions and/or inter-microbial interactions. Indeed, oral pathogens are not found as monospecies *in vivo*, but as mixed biofilm populations that are metabolically symbiotic (55, 56). Furthermore, studies have shown that some planktonic bacteria utilize SAM to produce the quorum sensor AI-2 for biofilm formation (57). If this metabolic system is also present in *Td*, this may explain why it is necessary to sense cytosolic concentrations of SAM and why the deletion of the CheR_like_ domain impairs chemotaxis and biofilm formation.

Chemotaxis assays unambiguously demonstrate that the CheR_like_ domain is solely responsible for the SAM chemoattractant response; deletion or mutation of the domain eliminates a response to SAM specifically. It is known that motility and chemotaxis play a role in *Td* biofilm formation and co-biofilm formation (19, 20). Therefore, chemoattraction toward SAM may also be involved in the formation of SAM-induced biofilms. The CheR_like_ domain likely binds to SAM at a surface-exposed cleft on its *C*-terminal subdomain. The solvent exposure of the ligand-binding site may allow for free diffusion of SAM within the cell, thus allowing the domain to sense current intracellular SAM concentrations. To date, this is the first reported instance of a characterized CheW-fused chemotaxis sensor. Although the mechanism for signal dispersal by the CheR_like_ domain to the chemotaxis array is unclear, our modeling suggests that the domain may interact with the CheA P5 regulatory domain and/or the CheA P4 catalytic domain. SAM-mediated signals may therefore be transmitted directly from the CheR_like_ domain to CheA through such interactions. Additionally, previous research has demonstrated that structural changes in CheW confer alterations to CheA activity (58, 59). It is, therefore, also possible that the presently proposed interface between the CheW and CheR_like_ domains of CheWS (or perhaps perturbations in the inter-domain linker) could modulate CheA activity.

Our bioinformatics analyses identified several classes of CheW proteins that possess additional fused domains. Two of these domains, chemoreceptor zinc-binding (CZB) and Per-ARNT-Sim (PAS) domains, are established sensor domains. CZB domains are known to act as hypochlorous acid (HOCl) sensors in a wide variety of proteins from diverse bacteria and can regulate protein activities in an HOCl-dependent manner (49, 60). PAS domains are present in both eukaryotes and prokaryotes and bind various ligands and/or proteins to modulate functionally diverse sensory pathways (61). Therefore, chemotactic sensing through CheW via a fused domain may be a widespread mechanism that allows bacteria to chemotactically respond to the current intracellular environment. Intriguingly, a recent report demonstrates that specific catecholamines are chemotactically sensed in *Vibrio campbellii* through direct binding to CheW (62). Together, these data show that the previous designation of CheW as a linker protein is a partial misnomer. In addition to stabilizing chemotaxis arrays, CheW is responsible for transmitting signals across the arrays and can function as an input site for ligand recognition. Due to these recent advancements, we propose to reclassify CheW as a “hub” protein that interplays signals from multiple proteins and/or ligands to derive a complete and functional chemotaxis array.

The fact that spirochetes have evolved and maintained a system to sense SAM indicates that the ligand is critical for cell survival, either as a nutrient or an important environmental cue. The most well-studied pathogenic spirochete is *Bb*, which is a tick-borne obligate parasite (35). Several studies in *Bb* show that SAM-mediated metabolic reactions are essential for cell growth and survival (35, 63). Furthermore, small molecules that prevent these reactions have been identified as suitable antimicrobial agents for *Bb* (63). In addition to being a necessary nutrient, metagenomics studies of tick microbiomes suggest that microbial SAM-mediated pathways may partly contribute to a more resilient microbiome (36). Although analogous research in *Td* has not yet been conducted, SAM-mediated pathways are present in all spirochetes (35). Determining if and how SAM is associated with *Td* pathogenesis in humans may greatly impact our current understanding of its virulence mechanisms.

In summary, we demonstrate an improved model for round body formation and identified a new class of protein sensors in spirochetes. Our results suggest that spirochetes may be more resilient than previously thought, as they continuously generate round bodies, even in favorable environments. A novel SAM-binding sensor, CheWS, influences round body formation, biofilm formation, and chemotaxis. Investigations into this system may reveal an alternative mechanism for chemotaxis signaling that occurs through CheW interactions and bypasses chemoreceptor-based inputs. Previous experiments have shown that some bacteria possess soluble chemotaxis arrays that are potentially responsible for sensing the current metabolic status of the cell (64). However, cryo-ET experiments in *Td* (30), *Bb* (65)*,* and *Tp* (66) do not show the presence of such soluble arrays. Indeed, chemotactic sensing via CheW may be functionally similar to soluble arrays and allow cells to sense the intracellular environment. We also show that SAM decreases the presence of round bodies and increases biofilm formation in *Td*. Importantly, it has been demonstrated that oral spirochete infections acquire resilience through chemotaxis and synergistic communal biofilms (54, 67). Collectively, our results exemplify the importance of examining non-canonical sensory systems in pathogenic bacteria, identify SAM as an influential factor in the life cycle of pathogenic spirochetes, and may offer new targets for antimicrobial therapies.

## MATERIALS AND METHODS

### Bacterial strains, culture conditions, and oligonucleotide primers

*T. denticola* ATCC 35405 (wild type) was used in this study. Cells were grown in tryptone-yeast extract-gelatin-volatile fatty acids-serum (TYGVS) medium at 37°C in an anaerobic chamber in the presence of 85% nitrogen, 10% carbon dioxide, and 5% hydrogen (68). For biofilm assay and growth curve analysis, *T. denticola* cells were grown in an oral bacterial growth medium (OBGM) (69). *T. denticola* Δ*cheR_like_* mutant and two point mutants, D321A, and D372A, were grown with erythromycin (50 µg/mL) (30). The oligonucleotide primers for PCR and reverse transcriptase PCR used in this study are listed in the table below. These primers were synthesized in IDT (Integrated DNA Technologies, Coralville, IA).

### Cryo-electron microscopy and tomography

*T. denticola* cells at the log phase and stationary phase were concentrated 50× via centrifugation at 3,000 × *g* for 5 minutes. For samples used for cryo-ET, protein A-treated 10-nm colloidal gold solution (Cell Microscopy Core, Utrecht University, The Netherlands) was added to a 1/10 dilution. For samples that contain *S*-adenosyl methionine (SAM), 0.5 mM SAM was added to the mixture and mixed by pipetting. For all samples, 3 µL aliquots of the mixture was applied to *R2*/2 200 mesh copper Quantiofoil grids (Quantifoil Micro Tools) that were freshly glow discharged. The grids were prepared using an automated Leica EM GP system (Leica Microsystems). The samples were pre-blotted for 60 seconds and blotted for 2 seconds in a chamber set at 95% humidity and 20°C. After blotting, the grids were immediately plunged frozen in liquid ethane.

Data collection for cryo-EM samples was conducted on a Talos transmission electron microscope operating at 120 kV. For cryo-ET experiments, data were collected on a Titan Krios transmission electron microscope (Thermo Fisher Scientific) operating at 300 kV equipped with a Gatan K3 Summit direct electron detector with a GIF Quantum energy filter (Gatan) at a slit width of 20 eV. Images were taken at a magnification of ×26,000, which corresponds to a pixel size of 3.27 Å. Defocus was set to −6 µm, and the total exposure was 100 e-/Å (2) per cell. Each tils series was collected with a bidirectional dose-symmetric tilt scheme with a 2° increment using Serial EM. Drift correcting and bread tracking–based tilt series alignment was done using IMOD (70). Contrast transfer function determination and correction were done using CTF plotter (71). Tomograms were reconstructive using simultaneous iterative reconstruction with binning set to 2 and iteration number set to 6.

### Protein purifications

DNA segments encoding the CheW-CheR_like_ protein in *T. denticola* were PCR amplified from *Td* genomic DNA using a forward oligonucleotide encoding an NdeI restriction site and a reverse primer encoding an EcoRI restriction site. The PCR products were treated with the appropriate restriction enzymes, purified, and ligated into a pet28a plasmid with a poly-Histidine tag and kanamycin resistance marker. Plasmids (pet28a) that encode the CheR_like_ domain and CheR_like_ domain mutant were purchased from GenScript. The plasmids were transformed into *Escherichia coli* BL21-DE3 cells and 4–8 L of cell culture were grown at 37°C until an optical density (OD) of 0.6 was reached. The flasks were cooled to 21°C and 1 mM of IPTG (isopropyl β-D-1-thiogalactopyranoside) was added to the culture. The cells were harvested after 16 hours of growth. The cells were lysed via sonication in lysis buffer (50 mM Tris pH 7.5, 150 mM NaCl, 5 mM imidazole) while cooled on ice. The lysate was centrifuged at 20,000 × *g* for 1 hour at 4°C. The lysate was then run over a gravity-flow purification column containing 3 mL of Nickel-NTA resin. The resin was washed with 10 mL of wash buffer (50 mM Tris pH 7.5, 150 mM NaCl, 20 mM imidazole), and the protein was eluted with 10 mL of elution buffer (50 mM Tris pH 7.5, 150 mM NaCl, 200 mM imidazole) and collected in 1-mL fractions. The fractions were assessed for protein concentration via Bradford reagent, and the fractions containing protein were run on a size-exclusion s75 and s200 column systems that monitored absorbance at 280 nm and collected 6-mL fractions. Fractions that contain the proteins were concentrated to ~10 mg/mL via centrifugation in a protein concentrator containing a regenerated cellulose filter with a 20-kDa molecular-weight cut-off. The protein solutions were aliquoted, flash-frozen in liquid nitrogen, and stored at −80°C.

### Isothermal calorimetry

Experiments were conducted on a TA Instruments Affinity ITC Low Volume calorimeter at 25°C. The proteins were purified in analysis buffer (50 mM Tris pH 7.5, 150 mM NaCl). *S*-adenosylmethionine (SAM) or *S*-adenosylhomocysteine (SAH) was also dissolved in the analysis buffer to 1 mM. Protein concentrations were determined by UV measurements using hypothetical absorption coefficients of the proteins. Two hundred microliters of ~200 µM protein was placed into the sample cell, and 200 µL of SAM or SAH was placed into a 250-µL syringe. Five microliter volumes of SAM or SAH were injected into the cell with 200 seconds between injections. The data were analyzed using NanoAnalyze v.3.11.0 with the Independent binding model. For all samples, heat exchanges that occur after SAM or SAH saturation were subtracted as a baseline from the titration data. Based on previous experiments (37, 72) the stoichiometry of SAM binding to CheR and CheW-R_like_ is 1:1. For data analyses on NanoAnalyze v.3.11.0, the active fraction of protein in the cell was varied to reflect the previously determined stoichiometric-binding ratio.

### Quantification of round body formation

Log phase *T. denticola* cells undergoing round body formation were quantified via cryo-EM microscopy. Grids containing the WT strain or *ΔcheR_like_* were prepared as described above and imaged on a Talos transmission electron microscope operating at 120 kV. For each sample, at least *n* = 113 cells were imaged and the percentage of cells undergoing round body formation was determined. For each strain, three separate cell cultures grown to the same OD_595_ were imaged and quantified with and without exogenous SAM added. The results are expressed as the mean percentage of cells forming round bodies ± 1 SD.

### Construction of two *TDE1492* site-directed mutants D321A and D372A

The vector shown in Fig. 6B was constructed to replace the wild-type *TDE1492* with two genes with site-directed mutations at amino acid D321 or D372 by using two-step PCR and DNA cloning, as previously documented (73) using primers listed in Table 1. In brief, to make this construct, the downstream region of *TDE1492* and the wild-type *TDE1492* were PCR amplified with primers P_1_/P_2_ and P_3_/P_4_, respectively, and then fused with primers P_1_/P_4_, generating Fragment 1, which was then cloned into the pMD19 T-vector (Takara Bio USA, Inc, Mountain View, CA). An erythromycin cassette (*ermB*) was PCR amplified with primers P_5_/P_6_ and cloned into the pGEM-T easy vector (Promega, Madison, WI). Fragment 1 and *ermB* were ligated together at a *Not*I cut site, generating *TDE1492-ermB*, which was used as a template to replace D321 and D372 with Ala, using primers P_7_/P_8_ and P_9_/P_10_. The resultant mutations were confirmed by DNA sequencing analysis. Site-directed mutagenesis was performed using Q5 Site-Directed Mutagenesis Kit (New England Biolabs, Ipswich, MA) according to the manufacturer’s instructions. The resultant plasmids, TDE1492*-ermB, were transformed into *T. denticola* wild-type competent cells via heat shock and then plated on soft agars with erythromycin, as previously described (4). Site-directed mutations were confirmed by PCR, followed by DNA sequencing.

**TABLE 1:** Oligonucleotide primers used in this study

Primers	Sequences (5′−3′)	Note^*a*^
P_1_	CGGGCGTAGGCATCGGAGATAC	TDE1492 mutation, 3′-end; [F]
P_2_	CATTTATTTCTCCTTATCCTTTTATATGCGGCCGCTTATTCTTTTGTAAAAATTAC	TDE1492 mutation, 3′-end; [R]
P_3_	GTAATTTTTACAAAAGAATAAGCGGCCGCATATAAAAGGATAAGGAGAAATAAATG	TDE1492 mutation; [F]
P_4_	CCCAGAGCACTTATCATAAC	TDE1492 mutation; [R]
P_5_	TCTAGACGATAGCTTCCGCTATTGC	Erythromycin cassette (*ermB*); [F]
P_6_	TCTAGATTTATCTACATTCCCTTTAGT AACG	Erythromycin cassette (*ermB*); [R]
P_7_	CTCATGTAAAGATTTATGCAAATGCTTCGGATTTGTTG	TDE1492 D321A mutation; [F]
P_8_	CACGAGGATAACGCATTTTTAATAG	TDE1492 D321A mutation; [R]
P_9_	CTTTTTGAGTACCATGCTTGTACTCATC	TDE1492 D372A mutation; [F]
P_10_	AATCATATCTTTTATCTCTTTTGAAAATGTC	TDE1492 D372A mutation; [R]
P_11_	ATGGAAGAAATGAAAGAAC	5′-end, confirm TDE1492 mutation; [F]
P_12_	GATATAGTTCTTGGCTCCAAG	3′-end, confirm TDE1492 mutation; [R]

^*a*^ Underlined sequences are engineered restriction cut sites for DNA cloning; [F] forward; [R] reverse.

### Chemotaxis assays

The chemotaxis of *T. denticola* was tested by capillary assay (30). Log-phase cultures of *T. denticola* were centrifuged at 5,000 × *g* for 7 minutes and supernatants were discarded. Cell pellets were resuspended in motility buffer (0.15 M NaCl, 10 mM NaH_2_PO_4_, 0.05 mM EDTA, 1% bovine serum albumin, and 0.5% methylcellulose). The motility buffer was equilibrated in an anaerobic chamber overnight. The final bacterial cell concentration was adjusted to 1 × 10^9^ cells/mL. Capillary tubes (0.025-mm inner diameter) were filled with either 0.5 mM hemin (positive control) or 0.5 mM SAM (New England Biolabs, Ipswich, MA) in the motility buffer and sealed with vacuum silicone grease (Dow Corning, catalogno. Z273554-1EA) before they were inserted into bacterial suspensions (500 µL each). After incubation in an anaerobic chamber at 37°C for 2 hours, the contents of each capillary tube were transferred to a new microcentrifuge tube and cell numbers were enumerated using a Petroff-Hausser counting chamber (Hausser Scientific, Horsham, PA). For the non-gradient control, capillary tubes were inserted into bacterial suspensions containing either 0.5 mM hemin, 0.5 mM SAM, or 0.5 mM SAH. The bacterial counts of each strain were normalized to those in the non-gradient control. Results are represented as the mean of cell numbers ± SDs.

### Biofilm formation assays

Biofilm formation was measured as previously described (74) with slight modifications. Briefly, 200 µL of mid-logarithmic-phase *T. denticola* cultures (10^8^ cells/mL) with or without 0.5 mM SAM was added into 96-well flat-bottom polystyrene plates. The plates were incubated anaerobically at 37°C for 7 days, allowing for biofilms to develop. The culture medium was carefully decanted, and the biofilms were stained with 150 µL of 0.1% crystal violet for 30 minutes, washed with water three times, and then air-dried. To quantify the amount of biofilms, 200 µL of 95% ethanol was added to each well and incubated for 30 minutes. The optical density at 595 nm (OD_595_) was measured using a Thermo Scientific Varioskan LUX multimode microplate reader (Bio-Rad). The results are represented as the average absorbance that is normalized to the WT strain without SAM addition ± SDs.

### Protein structural prediction and conservation analysis

The structure prediction of the *T. denticola* CheR_like_ domain from CheWS, full-length CheWS, and the *T. denticola* classical CheR protein were done using the Colab notebook (dpmd.ai/alphafold-colab) (42). Only residues 203–444 from CheWS were used, as these correspond to the CheR_like_ domain (30). The resulting models were examined, aligned, and compared to existing structures using PyMOL. The PyMOL script “Color h” was used to assess the hydrophobicity of the models according to the Eisenberg hydrophobicity scale. Residue conservation analysis was conducted using ConSurf (https://consurf.tau.ac.il). The sequences used in the ConSurf analyses are all previously identified spirochete CheRlike domains and F2 classical CheR proteins (30).

### Chemotaxis complex models

Molecular models were generated using AlphaFold-Multimer version 3.0 (42, 75) via the ColabFold (76) *AlphaFold2_mmseqs2* notebook at https://github.com/sokrypton/ColabFold.

### Domain architecture diversity of sequences with at least one CheW

We used the web portal of Pfam database v35.0 (77) to search for domain architectures containing the CheW Pfam model (PF01584). The database returns 111 unique domain architectures, including the single-domain CheW protein. There were 75 domain architectures that also contain at least one HATPase_c (PF02518), Hpt (PF01627), or both domains, which together with the CheW domain, are strong markers for sequences of CheA (28, 50). We removed them from the analysis by conservatively considering them CheA homologs. There were five domain architectures attributed to proteins that have been identified before, including CheWS (CheW-CheR_like_) in this study, which were also removed (CheV, 2XCheW, 3XCheW, and CheW-CZB). To our knowledge, the remaining 30 architectures have yet to be studied and show the versatility of the CheW domains in proteins with different biological functions. We classified these domain architectures according to their biological role using the categories in the MIST3 database list of signal transduction domains (50). These architectures and the classification are shown in Fig. S10.

## SUPPLEMENTAL MATERIAL

Figure S10 mbio.01598-23-s0001.pdf Bioinformatics results.

Supplemental material mbio.01598-23-s0002.pdf Fig. S1 to S9, Fig. S10 legend, and Table S1.

ASM does not own the copyrights to Supplemental Material that may be linked to, or accessed through, an article. The authors have granted ASM a non-exclusive, world-wide license to publish the Supplemental Material files. Please contact the corresponding author directly for reuse.

## References

[1] Wheatley RM , Poole PS . 2018. Mechanisms of bacterial attachment to roots. FEMS Microbiol Rev 42:448–461. doi:10.1093/femsre/fuy014 29672765

[2] Lux R , Miller JN , Park NH , Shi W . 2001. Motility and chemotaxis in tissue penetration of oral epithelial cell layers by Treponema denticola. Infect Immun 69:6276–6283. doi:10.1128/IAI.69.10.6276-6283.2001 11553571 PMC98762

[3] Sze CW , Zhang K , Kariu T , Pal U , Li C . 2012. Borrelia burgdorferi needs chemotaxis to establish infection in mammals and to accomplish its enzootic cycle. Infect Immun 80:2485–2492. doi:10.1128/IAI.00145-12 22508862 PMC3416460

[4] Alban PS , Johnson PW , Nelson DR . 2000. Serum-starvation-induced changes in protein synthesis and morphology of Borrelia burgdorferi. Microbiology (Reading) 146 ( Pt 1):119–127. doi:10.1099/00221287-146-1-119 10658658

[5] Meriläinen L , Herranen A , Schwarzbach A , Gilbert L . 2015. Morphological and biochemical features of Borrelia burgdorferi pleomorphic forms. Microbiology (Reading) 161:516–527. doi:10.1099/mic.0.000027 25564498 PMC4339653

[6] Umemoto T , Namikawa I , Yoshii Z , Konishi H . 1982. An internal view of the spherical body of Treponema macrodentium as revealed by scanning electron microscopy. Microbiol Immunol 26:191–198. doi:10.1111/j.1348-0421.1982.tb00171.x 7109978

[7] Leblhuber F , Huemer J , Steiner K , Gostner JM , Fuchs D . 2020. Knock-on effect of periodontitis to the pathogenesis of Alzheimer’s disease? Wien Klin Wochenschr 132:493–498. doi:10.1007/s00508-020-01638-5 32215721 PMC7519001

[8] Miklossy J . 2011. Alzheimer’s disease - a neurospirochetosis. analysis of the evidence following koch’s and hill’s criteria. J Neuroinflammation 8:1–16. doi:10.1186/1742-2094-8-90 21816039 PMC3171359

[9] Kamarajan P , Ateia I , Shin JM , Fenno JC , Le C , Zhan L , Chang A , Darveau R , Kapila YL . 2020. Periodontal pathogens promote cancer aggressivity via TLR/MyD88 triggered activation of integrin/FAK signaling that is therapeutically reversible by a probiotic bacteriocin. PLoS Pathog 16:e1008881. doi:10.1371/journal.ppat.1008881 33002094 PMC7529280

[10] Bamm VV , Ko JT , Mainprize IL , Sanderson VP , Wills MKB . 2019. Lyme disease frontiers: reconciling borrelia biology and clinical conundrums. Pathogens 8:1–59. doi:10.3390/pathogens8040299 PMC6963551 31888245

[11] Dunham-Ems SM , Caimano MJ , Eggers CH , Radolf JD . 2012. Borrelia burgdorferi requires the alternative sigma factor RpoS for dissemination within the vector during tick-to-mammal transmission. PLoS Pathog 8:e1002532. doi:10.1371/journal.ppat.1002532 22359504 PMC3280991

[12] Brorson Ø , Brorson SH . 1998. In vitro conversion of Borrelia burgdorferi to cystic forms in spinal fluid, and transformation to mobile spirochetes by incubation in BSK-H medium. Infection 26:144–150. doi:10.1007/BF02771839 9646104

[13] Brorson O , Brorson SH . 1998. A rapid method for generating cystic forms of Borrelia burgdorferi, and their reversal to mobile spirochetes. APMIS 106:1131–1141. doi:10.1111/j.1699-0463.1998.tb00269.x 10052721

[14] Arrazuria R , Caddey B , Cobo ER , Barkema HW , De Buck J . 2021. Effects of different culture media on growth of Treponema spp. isolated from digital dermatitis. Anaerobe 69:102345. doi:10.1016/j.anaerobe.2021.102345 33596466

[15] Feng J , Auwaerter PG , Zhang Y , Brissette CA . 2015. Drug combinations against Borrelia burgdorferi Persisters in vitro: eradication achieved by using daptomycin, cefoperazone and doxycycline. PLoS ONE 10:e0117207. doi:10.1371/journal.pone.0117207 25806811 PMC4373819

[16] Feng J , Shi W , Zhang S , Sullivan D , Auwaerter PG , Zhang Y . 2016. A drug combination screen identifies drugs active against amoxicillin-induced round bodies of in vitro Borrelia burgdorferi persisters from an FDA drug library. Front Microbiol 7:743. doi:10.3389/fmicb.2016.00743 27242757 PMC4876775

[17] Rudenko N , Golovchenko M , Kybicova K , Vancova M . 2019. Metamorphoses of Lyme disease spirochetes: phenomenon of borrelia persisters. Parasit Vectors 12:237. doi:10.1186/s13071-019-3495-7 31097026 PMC6521364

[18] MacDonald AB . 2006. Plaques of Alzheimer’s disease originate from cysts of Borrelia burgdorferi, the Lyme disease spirochete. Medical Hypotheses 67:592–600. doi:10.1016/j.mehy.2006.02.035 16675154

[19] Vesey PM , Kuramitsu HK . 2004. Genetic analysis of Treponema denticola ATCC 35405 biofilm formation. Microbiology (Reading) 150:2401–2407. doi:10.1099/mic.0.26816-0 15256581

[20] Ng HM , Slakeski N , Butler CA , Veith PD , Chen Y-Y , Liu SW , Hoffmann B , Dashper SG , Reynolds EC . 2019. The role of Treponema Denticola motility in synergistic biofilm formation with Porphyromonas gingivalis. Front Cell Infect Microbiol 9:1–15. doi:10.3389/fcimb.2019.00432 31921707 PMC6930189

[21] Huang Z , Wang Y-H , Zhu H-Z , Andrianova EP , Jiang C-Y , Li D , Ma L , Feng J , Liu Z-P , Xiang H , Zhulin IB , Liu S-J . 2019. Cross talk between chemosensory pathways that modulate chemotaxis and biofilm formation. mBio 10:1–15. doi:10.1128/mBio.02876-18 PMC6391922 30808696

[22] Briegel A , Li X , Bilwes AM , Hughes KT , Jensen GJ , Crane BR . 2012. Bacterial chemoreceptor arrays are hexagonally packed trimers of receptor dimers networked by rings of kinase and coupling proteins. Proc Natl Acad Sci U S A 109:3766–3771. doi:10.1073/pnas.1115719109 22355139 PMC3309718

[23] Hazelbauer GL , Falke JJ , Parkinson JS . 2008. Bacterial chemoreceptors: high-performance signaling in networked arrays. Trends Biochem Sci 33:9–19. doi:10.1016/j.tibs.2007.09.014 18165013 PMC2890293

[24] Yang W , Cassidy CK , Ames P , Diebolder CA , Schulten K , Luthey-Schulten Z , Parkinson JS , Briegel A . 2019. In situ conformational changes of the Escherichia coli Serine chemoreceptor in different signaling states. mBio 10:1–14. doi:10.1128/mBio.00973-19 PMC6606802 31266867

[25] Muok AR , Briegel A , Crane BR . 2020. Regulation of the chemotaxis histidine kinase CheA: a structural perspective. Biochim Biophys Acta Biomembr 1862:183030. doi:10.1016/j.bbamem.2019.183030 31374212 PMC7212787

[26] Xu H , Sultan S , Yerke A , Moon KH , Wooten RM , Motaleb MA . 2017. Borrelia burgdorferi CheY2 is dispensable for chemotaxis or motility but crucial for the infectious life cycle of the spirochete. Infect Immun 85:1–14. doi:10.1128/IAI.00264-16 PMC5203640 27799336

[27] Moon KH , Hobbs G , Motaleb MA . 2016. Borrelia burgdorferi CheD promotes various functions in chemotaxis and the pathogenic life cycle of the spirochete. Infect Immun 84:1743–1752. doi:10.1128/IAI.01347-15 27021244 PMC4907153

[28] Wuichet K , Zhulin IB . 2010. Origins and diversification of a complex signal transduction system in prokaryotes. Sci Signal 3:1–27. doi:10.1126/scisignal.2000724 PMC3401578 20587806

[29] Su X , Tang Z , Lu Z , Liu Y , He W , Jiang J , Zhang Y , Wu H . 2021. Oral Treponema denticola infection induces Aβ1–40 and Aβ1–42 accumulation in the hippocampus of C57BL/6 mice. J Mol Neurosci 71:1506–1514. doi:10.1007/s12031-021-01827-5 33763842

[30] Muok AR , Ortega DR , Kurniyati K , Yang W , Maschmann ZA , Sidi Mabrouk A , Li C , Crane BR , Briegel A . 2020. Atypical chemoreceptor arrays accommodate high membrane curvature. Nat Commun 11:5763. doi:10.1038/s41467-020-19628-6 33188180 PMC7666581

[31] Zhang K , Liu J , Tu Y , Xu H , Charon NW , Li C . 2012. Two CheW coupling proteins are essential in a chemosensory pathway of Borrelia burgdorferi. Mol Microbiol 85:782–794. doi:10.1111/j.1365-2958.2012.08139.x 22780444 PMC3418435

[32] Djordjevic S , Stock AM . 1997. Crystal structure of the chemotaxis receptor methyltransferase CheR suggests a conserved structural motif for binding S-adenosylmethionine. Structure 5:545–558. doi:10.1016/s0969-2126(97)00210-4 9115443

[33] Sourjik V , Berg HC . 2002. Receptor sensitivity in bacterial chemotaxis. Proc Natl Acad Sci U S A 99:123–127. doi:10.1073/pnas.011589998 11742065 PMC117525

[34] Lin H . 2011. S-adenosylmethionine-dependent alkylation reactions: when are radical reactions used? Bioorg Chem 39:161–170. doi:10.1016/j.bioorg.2011.06.001 21762947 PMC3188380

[35] Parveen N , Cornell KA . 2011. Methylthioadenosine/S-adenosylhomocysteine nucleosidase, a critical enzyme for bacterial metabolism. Mol Microbiol 79:7–20. doi:10.1111/j.1365-2958.2010.07455.x 21166890 PMC3057356

[36] Obregón D , Bard E , Abrial D , Estrada-Peña A , Cabezas-Cruz A . 2019. Sex-specific linkages between taxonomic and functional profiles of tick gut microbiomes. Front Cell Infect Microbiol 9:298. doi:10.3389/fcimb.2019.00298 31475121 PMC6702836

[37] Djordjevic S , Stock AM . 1998. Chemotaxis receptor recognition by protein methyltransferase CheR. Nat Struct Biol 5:446–450. doi:10.1038/nsb0698-446 9628482

[38] Wolf V , Lange R , Wecke J . 1993. Development of quasi-multicellular bodies of Treponema denticola. Arch Microbiol 160:206–213. doi:10.1007/BF00249126 8215797

[39] Margulis L , Maniotis A , MacAllister J , Scythes J , Brorson O , Hall J , Krumbein WE , Chapman MJ . 2009. Spirochete round bodies, syphilis, Lyme disease & AIDS: resurgence of ‘the great Imitator. Symbiosis 47:51–58. doi:10.1007/BF03179970

[40] DeLamater ED , Wiggall RH , Haanes M . 1950. Studies on the life cycle of spirochetes. J Exp Med 92:239–246. doi:10.1084/jem.92.3.239 15436933 PMC2136033

[41] Muok AR , Deng Y , Gumerov VM , Chong JE , DeRosa JR , Kurniyati K , Coleman RE , Lancaster KM , Li C , Zhulin IB , Crane BR . 2019. A di-iron protein recruited as an Fe[II] and oxygen sensor for bacterial chemotaxis functions by stabilizing an iron-peroxy species. Proc Natl Acad Sci U S A 116:14955–14960. doi:10.1073/pnas.1904234116 31270241 PMC6660769

[42] Jumper J , Evans R , Pritzel A , Green T , Figurnov M , Ronneberger O , Tunyasuvunakool K , Bates R , Žídek A , Potapenko A , Bridgland A , Meyer C , Kohl SAA , Ballard AJ , Cowie A , Romera-Paredes B , Nikolov S , Jain R , Adler J , Back T , Petersen S , Reiman D , Clancy E , Zielinski M , Steinegger M , Pacholska M , Berghammer T , Bodenstein S , Silver D , Vinyals O , Senior AW , Kavukcuoglu K , Kohli P , Hassabis D . 2021. Highly accurate protein structure prediction with Alphafold. Nature 596:583–589. doi:10.1038/s41586-021-03819-2 34265844 PMC8371605

[43] Batra M , Sharma R , Malik A , Dhindwal S , Kumar P , Tomar S . 2016. Crystal structure of pentapeptide-independent chemotaxis receptor methyltransferase (CheR) reveals idiosyncratic structural determinants for receptor recognition. J Struct Biol 196:364–374. doi:10.1016/j.jsb.2016.08.005 27544050

[44] Cassidy CK , Himes BA , Sun D , Ma J , Zhao G , Parkinson JS , Stansfeld PJ , Luthey-Schulten Z , Zhang P . 2020. Structure and dynamics of the E. coli chemotaxis core signaling complex by cryo-electron tomography and molecular simulations. Commun Biol 3:24. doi:10.1038/s42003-019-0748-0 31925330 PMC6954272

[45] Cassidy CK , Himes BA , Alvarez FJ , Ma J , Zhao G , Perilla JR , Schulten K , Zhang P . 2015. CryoEM and computer simulations reveal a novel kinase conformational switch in bacterial chemotaxis signaling. Elife 4:1–20. doi:10.7554/eLife.08419 PMC6746300 26583751

[46] Muok AR , Chua TK , Srivastava M , Yang W , Maschmann Z , Borbat PP , Chong J , Zhang S , Freed JH , Briegel A , Crane BR . 2020. Engineered chemotaxis core signaling units indicate a constrained kinase-off state. Sci Signal 13:1–14. doi:10.1126/scisignal.abc1328 PMC7790435 33172954

[47] Fredrick KL , Helmann JD . 1994. Dual Chemotaxis signaling pathways in Bacillus subtilis: A Σ(D)-dependent gene encodes a novel protein with both CheW and CheY homologous domains. J Bacteriol 176:2727–2735. doi:10.1128/jb.176.9.2727-2735.1994 8169223 PMC205414

[48] Briegel A , Ortega DR , Mann P , Kjær A , Ringgaard S , Jensen GJ . 2016. Chemotaxis cluster 1 proteins form cytoplasmic arrays in Vibrio cholerae and are stabilized by a double signaling domain receptor DosM. Proc Natl Acad Sci U S A 113:10412–10417. doi:10.1073/pnas.1604693113 27573843 PMC5027440

[49] Perkins A , Tudorica DA , Teixeira RD , Schirmer T , Zumwalt L , Ogba OM , Cassidy CK , Stansfeld PJ , Guillemin K . 2021. A bacterial inflammation sensor regulates c-di-GMP signaling, adhesion, and biofilm formation. mBio 12:e0017321. doi:10.1128/mBio.00173-21 34154415 PMC8262984

[50] Gumerov VM , Ortega DR , Adebali O , Ulrich LE , Zhulin IB . 2020. MiST 3.0: an updated microbial signal transduction database with an emphasis on chemosensory systems. Nucleic Acids Res 48:D459–D464. doi:10.1093/nar/gkz988 31754718 PMC6943060

[51] Briegel A , Jensen G . 2017. Progress and potential of electron cryotomography as illustrated by its application to bacterial chemoreceptor arrays. Annu Rev Biophys 46:1–21. doi:10.1146/annurev-biophys-070816-033555 28301773

[52] Jutras BL , Scott M , Parry B , Biboy J , Gray J , Vollmer W , Jacobs-Wagner C . 2016. Lyme disease and relapsing fever Borrelia elongate through zones of Peptidoglycan synthesis that mark division sites of daughter cells. Proc Natl Acad Sci U S A 113:9162–9170. doi:10.1073/pnas.1610805113 27506799 PMC4995948

[53] Blanco DR , Reimann K , Skare J , Champion CI , Foley D , Exner MM , Hancock RE , Miller JN , Lovett MA . 1994. Isolation of the outer membranes from Treponema pallidum and Treponema vincentii. J Bacteriol 176:6088–6099. doi:10.1128/jb.176.19.6088-6099.1994 7928971 PMC196829

[54] Zhu Y , Dashper SG , Chen Y-Y , Crawford S , Slakeski N , Reynolds EC , Brissette CA . 2013. Porphyromonas gingivalis and Treponema denticola synergistic polymicrobial biofilm development. PLoS ONE 8:e71727. doi:10.1371/journal.pone.0071727 23990979 PMC3753311

[55] Slots J , Genco RJ . 1984. Black-pigmented Bacteroides species, Capnocytophaga species, and Actinobacillus actinomycetemcomitans in human periodontal disease: virulence factors in colonization, survival, and tissue destruction. J Dent Res 63:412–421. doi:10.1177/00220345840630031101 6583243

[56] Mark Welch JL , Rossetti BJ , Rieken CW , Dewhirst FE , Borisy GG . 2016. Biogeography of a human oral microbiome at the micron scale. Proc Natl Acad Sci U S A 113:E791–800. doi:10.1073/pnas.1522149113 26811460 PMC4760785

[57] Wang Y , Bian Z , Wang Y . 2022. Biofilm formation and inhibition mediated by bacterial quorum sensing. Appl Microbiol Biotechnol 106:6365–6381. doi:10.1007/s00253-022-12150-3 36089638

[58] Piñas GE , Frank V , Vaknin A , Parkinson JS . 2016. The source of high signal cooperativity in bacterial chemosensory arrays. Proc Natl Acad Sci U S A 113:3335–3340. doi:10.1073/pnas.1600216113 26951681 PMC4812747

[59] Ortega DR , Mo G , Lee K , Zhou H , Baudry J , Dahlquist FW , Zhulin IB . 2013. Conformational coupling between receptor and kinase binding sites through a conserved salt bridge in a signaling complex scaffold protein. PLoS Comput Biol 9:e1003337. doi:10.1371/journal.pcbi.1003337 24244143 PMC3828127

[60] Perkins A , Tudorica DA , Amieva MR , Remington SJ , Guillemin K . 2019. Helicobacter Pylori senses bleach as a chemoattractant using a cytosolic chemoreceptor. PLoS Biol 17:e3000395. doi:10.1371/journal.pbio.3000395 31465435 PMC6715182

[61] Henry JT , Crosson S . 2011. Ligand-binding PAS domains in a genomic, cellular, and structural context. Annu Rev Microbiol 65:261–286. doi:10.1146/annurev-micro-121809-151631 21663441 PMC3298442

[62] Weigert Muñoz A , Hoyer E , Schumacher K , Grognot M , Taute K , Hacker S , Sieber S , Jung K . 2021. Eukaryotic catecholamine hormones influence the Chemotactic control of Vibrio Campbellii by binding to the coupling protein chew. Chemistry. doi:10.26434/chemrxiv-2021-gx4mr PMC8915975 35238645

[63] Cornell KA , Primus S , Martinez JA , Parveen N . 2009. Assessment of methylthioadenosine/S-adenosylhomocysteine nucleosidases of Borrelia burgdorferi as targets for novel antimicrobials using a novel high-throughput method. J Antimicrob Chemother 63:1163–1172. doi:10.1093/jac/dkp129 19376840 PMC2734086

[64] Briegel A , Ladinsky MS , Oikonomou C , Jones CW , Harris MJ , Fowler DJ , Chang Y-W , Thompson LK , Armitage JP , Jensen GJ . 2014. Structure of bacterial cytoplasmic chemoreceptor arrays and implications for chemotactic signaling. Elife 3:e02151. doi:10.7554/eLife.02151 24668172 PMC3964821

[65] Xu H , Raddi G , Liu J , Charon NW , Li C . 2011. Chemoreceptors and Flagellar motors are subterminally located in close proximity at the two cell poles in spirochetes. J Bacteriol 193:2652–2656. doi:10.1128/JB.01530-10 21441520 PMC3133147

[66] Liu J , Howell JK , Bradley SD , Zheng Y , Zhou ZH , Norris SJ . 2010. Cellular architecture of Treponema pallidum: novel flagellum, periplasmic cone, and cell envelope as revealed by cryo electron tomography. J Mol Biol 403:546–561. doi:10.1016/j.jmb.2010.09.020 20850455 PMC2957517

[67] Yamada M , Ikegami A , Kuramitsu HK . 2005. Synergistic biofilm formation by Treponema denticola and Porphyromonas gingivalis. FEMS Microbiol Lett 250:271–277. doi:10.1016/j.femsle.2005.07.019 16085371

[68] Ohta K , Makinen KK , Loesche WJ . 1986. Purification and characterization of an enzyme produced by Treponema denticola capable of hydrolyzing synthetic trypsin substrates. Infect Immun 53:213–220. doi:10.1128/iai.53.1.213-220.1986 3013780 PMC260099

[69] Orth R , O’Brien-Simpson N , Dashper S , Walsh K , Reynolds E . 2010. An efficient method for enumerating oral spirochetes using flow cytometry. J Microbiol Methods 80:123–128. doi:10.1016/j.mimet.2009.11.006 19932718

[70] Mastronarde DN . 1997. Dual-axis tomography: an approach with alignment methods that preserve resolution. J Struct Biol 120:343–352. doi:10.1006/jsbi.1997.3919 9441937

[71] Xiong Q , Morphew MK , Schwartz CL , Hoenger AH , Mastronarde DN . 2009. CTF determination and correction for low dose tomographic tilt series. J Struct Biol 168:378–387. doi:10.1016/j.jsb.2009.08.016 19732834 PMC2784817

[72] García-Fontana C , Reyes-Darias JA , Muñoz-Martínez F , Alfonso C , Morel B , Ramos JL , Krell T . 2013. High specificity in CheR methyltransferase function. J Biol Chem 288:18987–18999. doi:10.1074/jbc.M113.472605 23677992 PMC3696673

[73] Kurniyati K , Li C . 2016. pyrF as a counterselectable marker for unmarked genetic manipulations in Treponema denticola. Appl Environ Microbiol 82:1346–1352. doi:10.1128/AEM.03704-15 26682856 PMC4751821

[74] Bian J , Liu X , Cheng YQ , Li C . 2013. Inactivation of cyclic di-GMP binding protein TDE0214 affects the motility, biofilm formation, and virulence of Treponema denticola. J Bacteriol 195:3897–3905. doi:10.1128/JB.00610-13 23794624 PMC3754597

[75] Evans R , O’Neill M , Pritzel A , Antropova N , Senior A , Green T , Žídek A , Bates R , Blackwell S , Yim J , Ronneberger O , Bodenstein S , Zielinski M , Bridgland A , Potapenko A , Cowie A , Tunyasuvunakool K , Jain R , Clancy E , Kohli P , Jumper J , Hassabis D . 2021. Protein complex prediction with Alphafold-Multimer. bioRxiv. doi:10.1101/2021.10.04.463034

[76] Mirdita M , Schütze K , Moriwaki Y , Heo L , Ovchinnikov S , Steinegger M . 2022. ColabFold: making protein folding accessible to all. Nat Methods 19:679–682. doi:10.1038/s41592-022-01488-1 35637307 PMC9184281

[77] Mistry J , Finn RD , Eddy SR , Bateman A , Punta M . 2013. Challenges in homology search: HMMER3 and convergent evolution of coiled-coil regions. Nucleic Acids Res 41:1–10. doi:10.1093/nar/gkt263 23598997 PMC3695513

