# Supplementary figures and images for: A new class of protein sensor links spirochete pleomorphism, persistence, and chemotaxis
Source: mBio. 2023 Aug 21;14(5):e01598-23. doi: 10.1128/mbio.01598-23 (PMC10653840; doi:10.1128/mbio.01598-23)

input

transmitter

receiver-transmitter

receiver

unknown

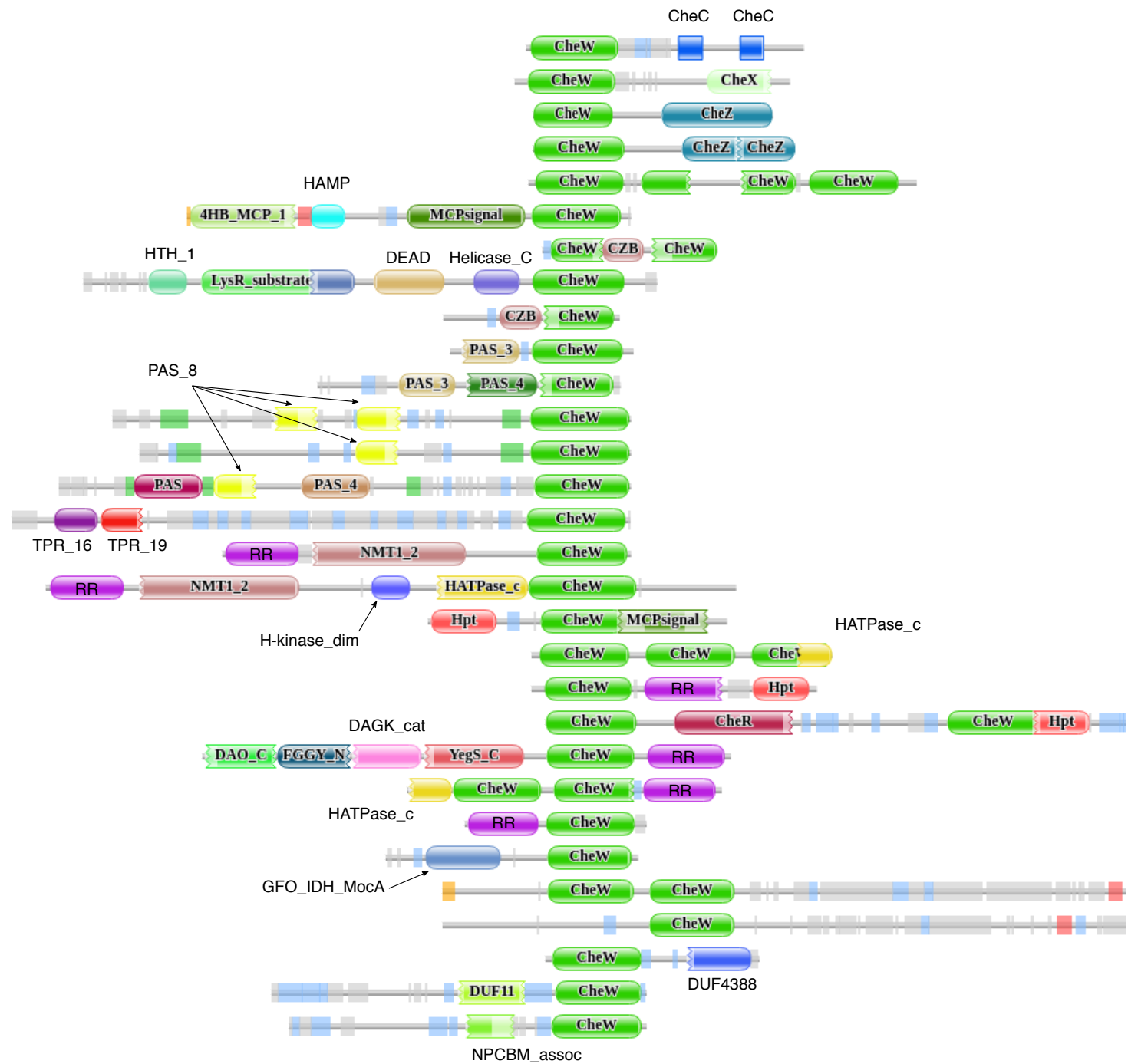

Supplement: Figure S10 — Bioinformatics results. [file mbio.01598-23-s0001.pdf]
