# Supplementary material for: A new class of protein sensor links spirochete pleomorphism, persistence, and chemotaxis
Source: mBio. 2023 Aug 21;14(5):e01598-23. doi: 10.1128/mbio.01598-23 (PMC10653840; doi:10.1128/mbio.01598-23)
Supplement: Supplemental material — Fig. S1 to S9, Fig. S10 legend, and Table S1. [file mbio.01598-23-s0002.pdf]

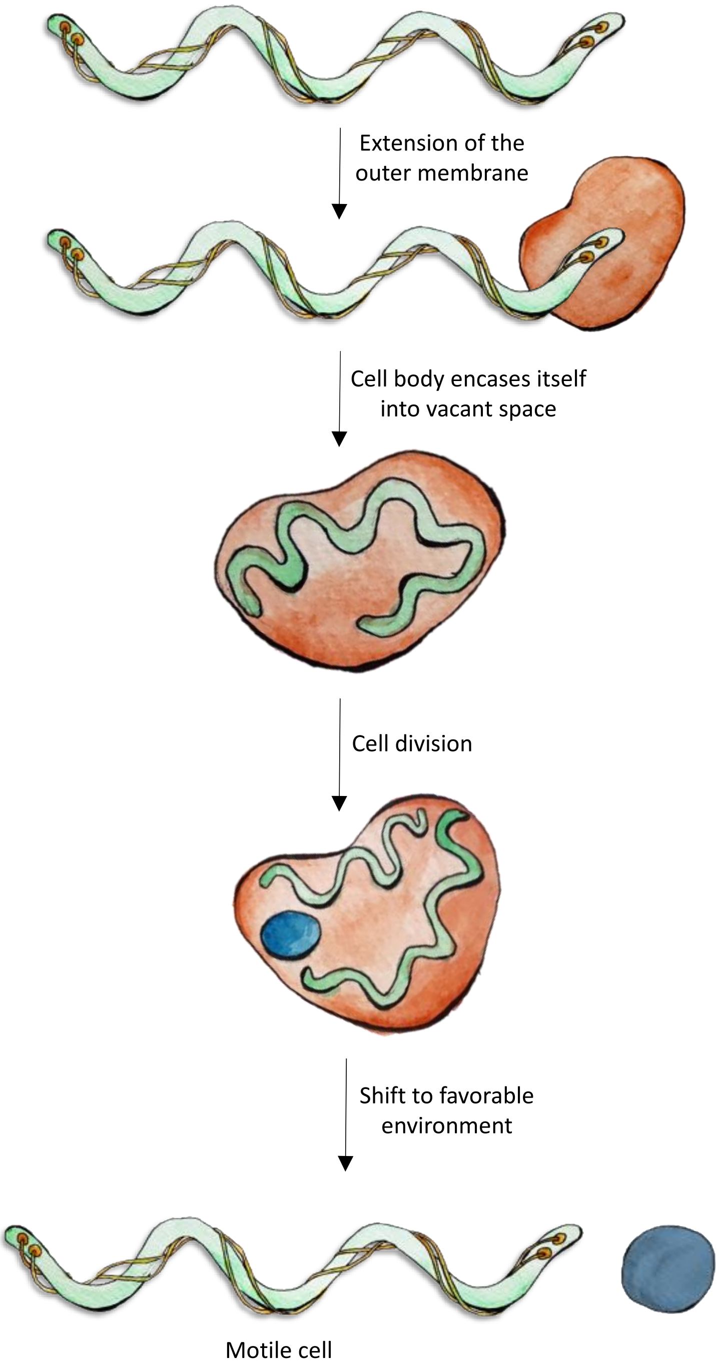

Fig S1: The established model for round body formation.
Previous microscopy experiments in *Td, Tp,* and *Bb* have led to an established model for round body formation. In summary, unfavorable conditions induce the outer membrane to enlarge at the cell tip so that the spiral-shaped spirochete can move into the vacant space. Here, the cells are protected and can divide into more spiral cells or 'core structures' (dark blue). When the conditions are favorable, the cells are released and the spiral cells are motile. The core structures may take several weeks to form motile spiral cells.

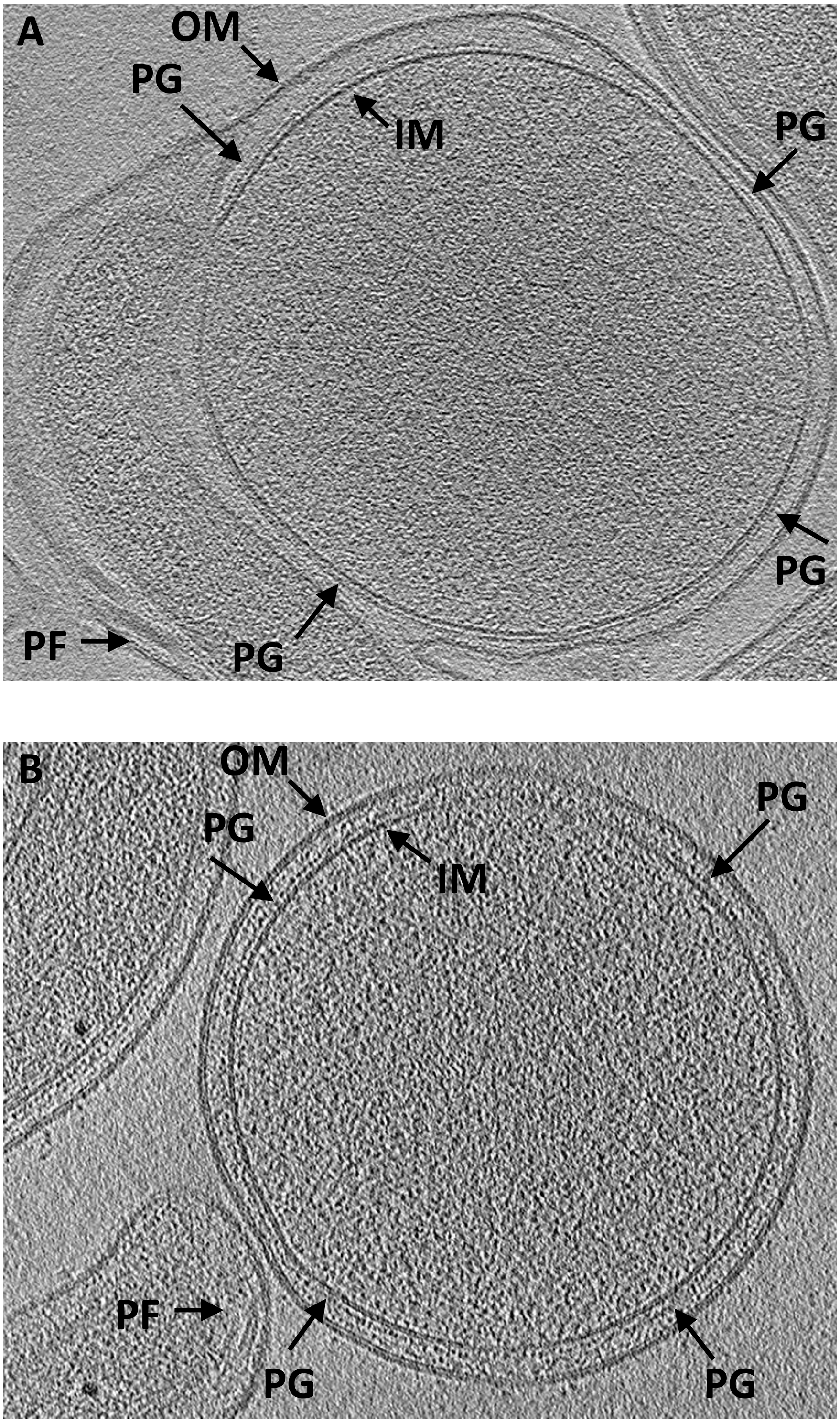

Fig. S2: The peptidoglycan (PG) layer in log phase round bodies.
(A) Log phase round bodies possess a continuous PG layer during their formation and **(B)** after separation from the spiral cells. In both stages, the cell outer membrane (OM), inner membrane (IM), and periplasmic flagella (PF) are also visible.

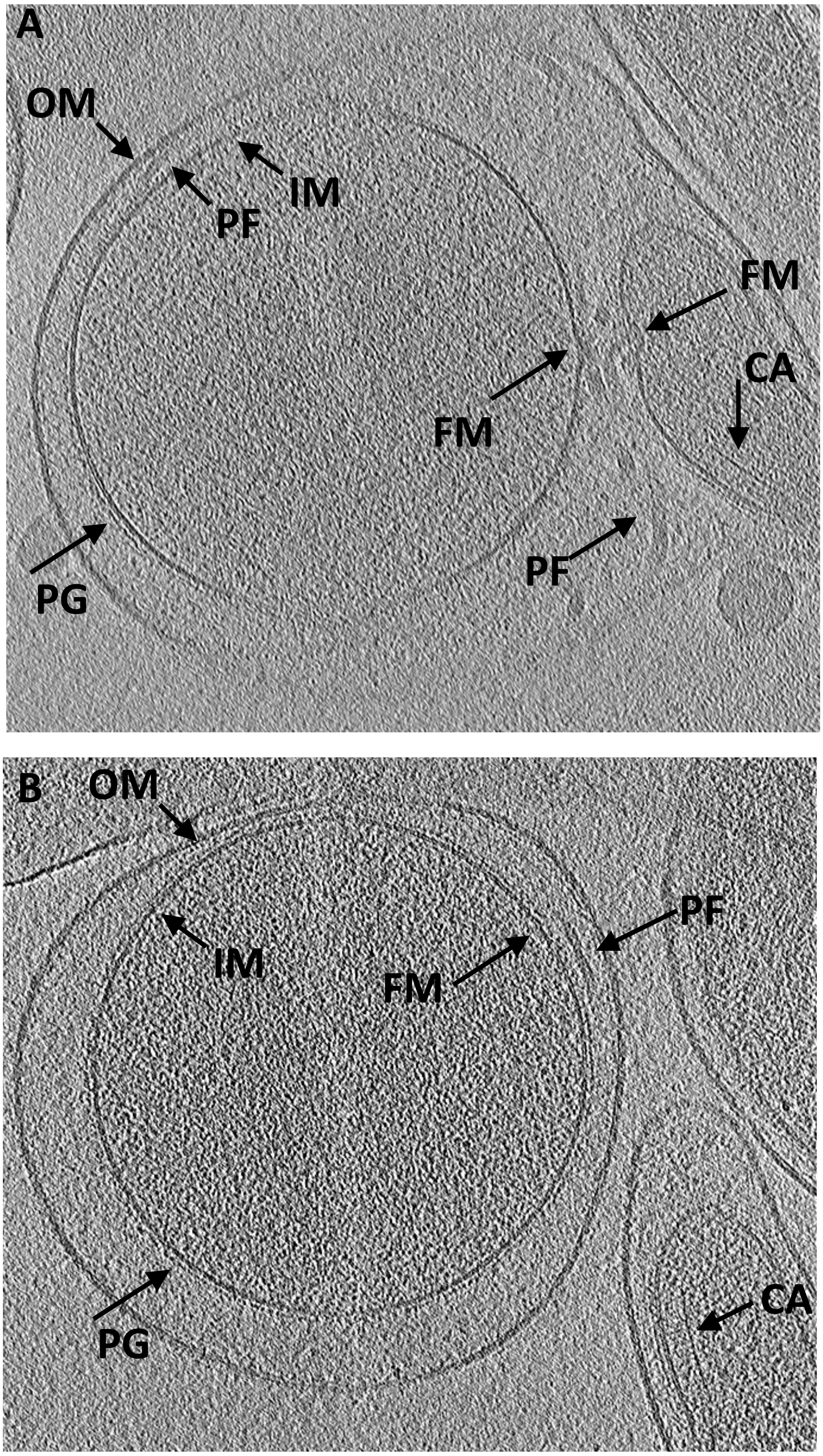

Fig. S3: Motility machinery is present in log phase round bodies.
(A) During their formation, periplasmic flagella (F) and flagellar motors (FM) are attached to round bodies. The round bodies do not possess visible chemotaxis arrays (CA) but arrays are seen in the spiral cells near the forming round body. **(B)** After separation, round bodies can still possess periplasmic flagella and flagellar motors. At both stages, the outer membrane (OM) and inner membrane (IM) remain intact.

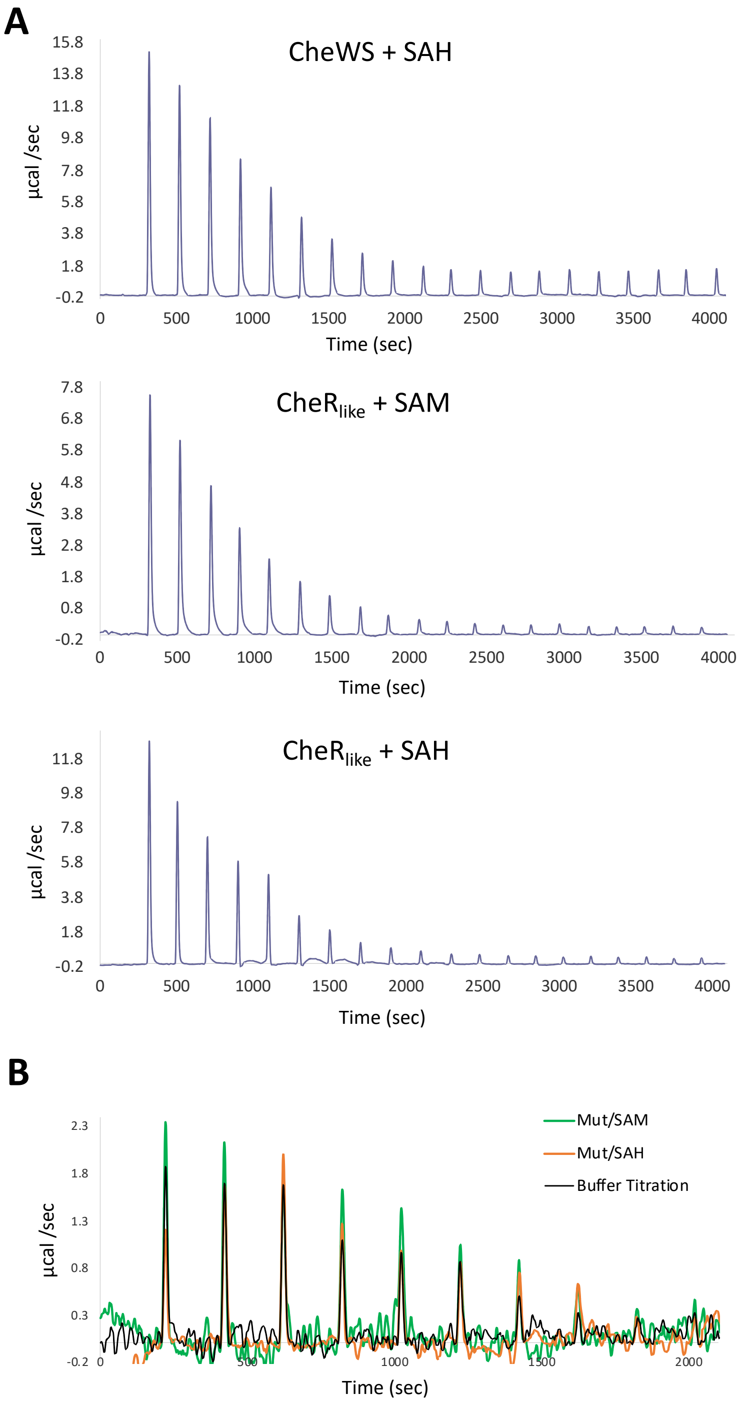

Fig S4 Output from isothermal calorimetry experiments.
(A) Previous Isothermal calorimetry experiments with CheWS demonstrate that the protein binds SAM with a kd of ~9 µM[29]. Here we show that CheWS binds SAH with a Kd of ~17 µM. The isolated $CheR_{like}$ domain binds SAM and SAH with a Kd of 22 µM and 35 µM, respectively. **(B)** A $CheR_{like}$ mutant that is altered in the hypothetical ligand site (E297A, D321A) does not bind SAM or SAH, and produces data consistent with buffer titrations.

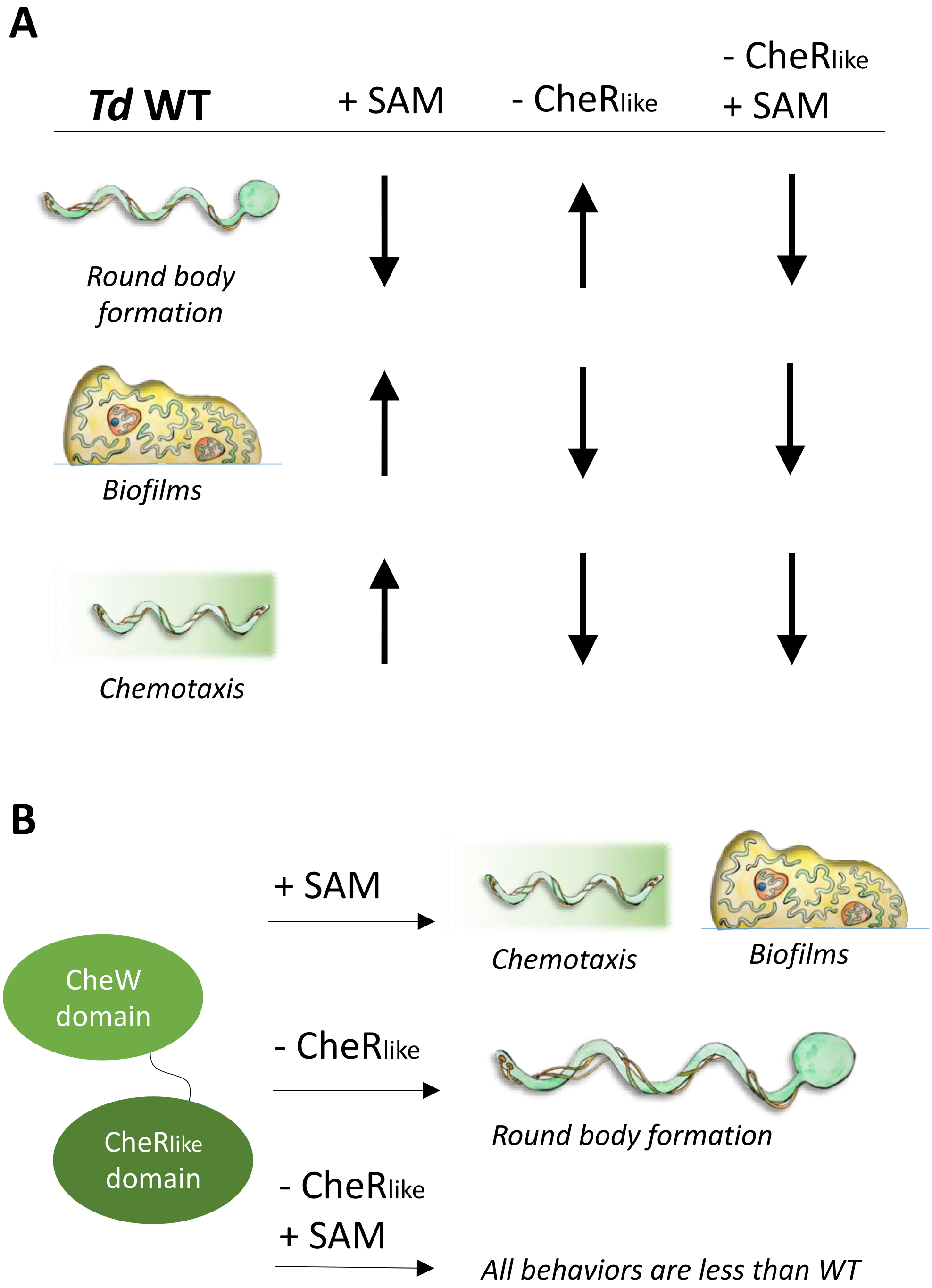

Fig. S5: A graphical summary of the round body quantifications, biofilm quantifications, and chemotaxis assays.

(A) Compared to WT cells without exogenous SAM added, the addition of SAM decreases round body formation, increases biofilm formation, and enables chemotaxis toward SAM. When the $CheR_{like}$ domain is absent from the cells, the exact opposite trends are produced. When the $CheR_{like}$ domain is absent and exogenous SAM is present, all behaviors are reduced when compared to WT without SAM. **(B)** The $CheR_{like}$ domain enables chemotaxis toward SAM and biofilm formation. When the domain is absent, chemotaxis and biofilm formation are suppressed, but more cells undergo round body formation. When the domain is absent and SAM is present, all behaviors are relatively suppressed.

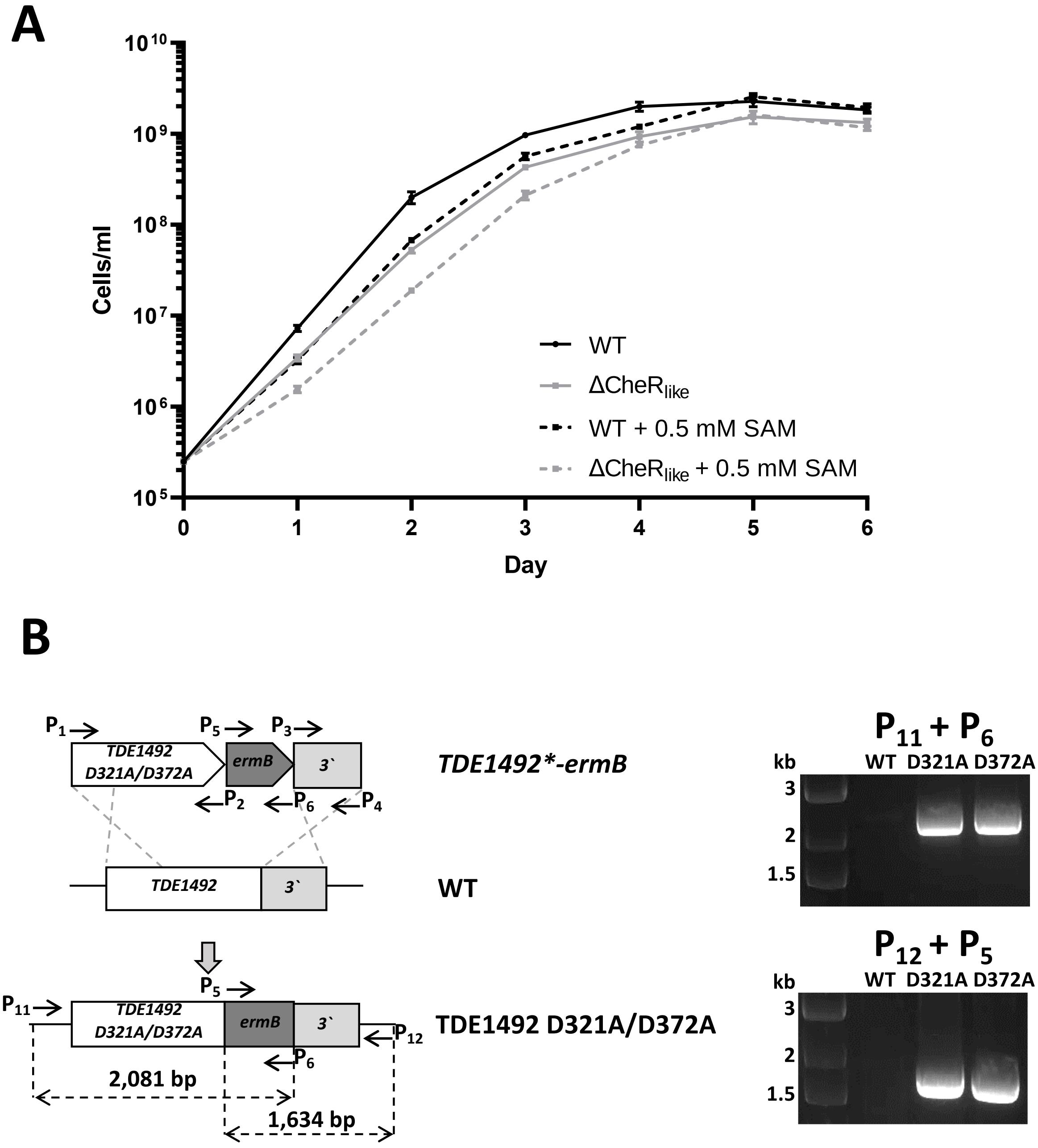

Fig. S6. Growth and engineering of *Td* strains.
(**A**) Planktonic growth curves of the strains used in these experiments, with and without the addition of SAM, show that planktonic growth does not differ at statioary growth phase. (**B)** A diagram showing the construction of two site-directed mutants, D321A and D372A in *T. denticola*. Amplification of mutated gene by PCR. The vectors (*TDE1492**-ermB*) (top panel) with the mutations (D321A or D372A) were constructed and transformed into *T. denticola* WT (middle panel). The resultant mutants (lower panel) were confirmed by PCR, followed by DNA sequencing. Arrows represent the relative positions and orientations of these primers which are listed in Table 1. The numbers (bp) are predicted the sizes of PCR products using the corresponding primers as illustrated.

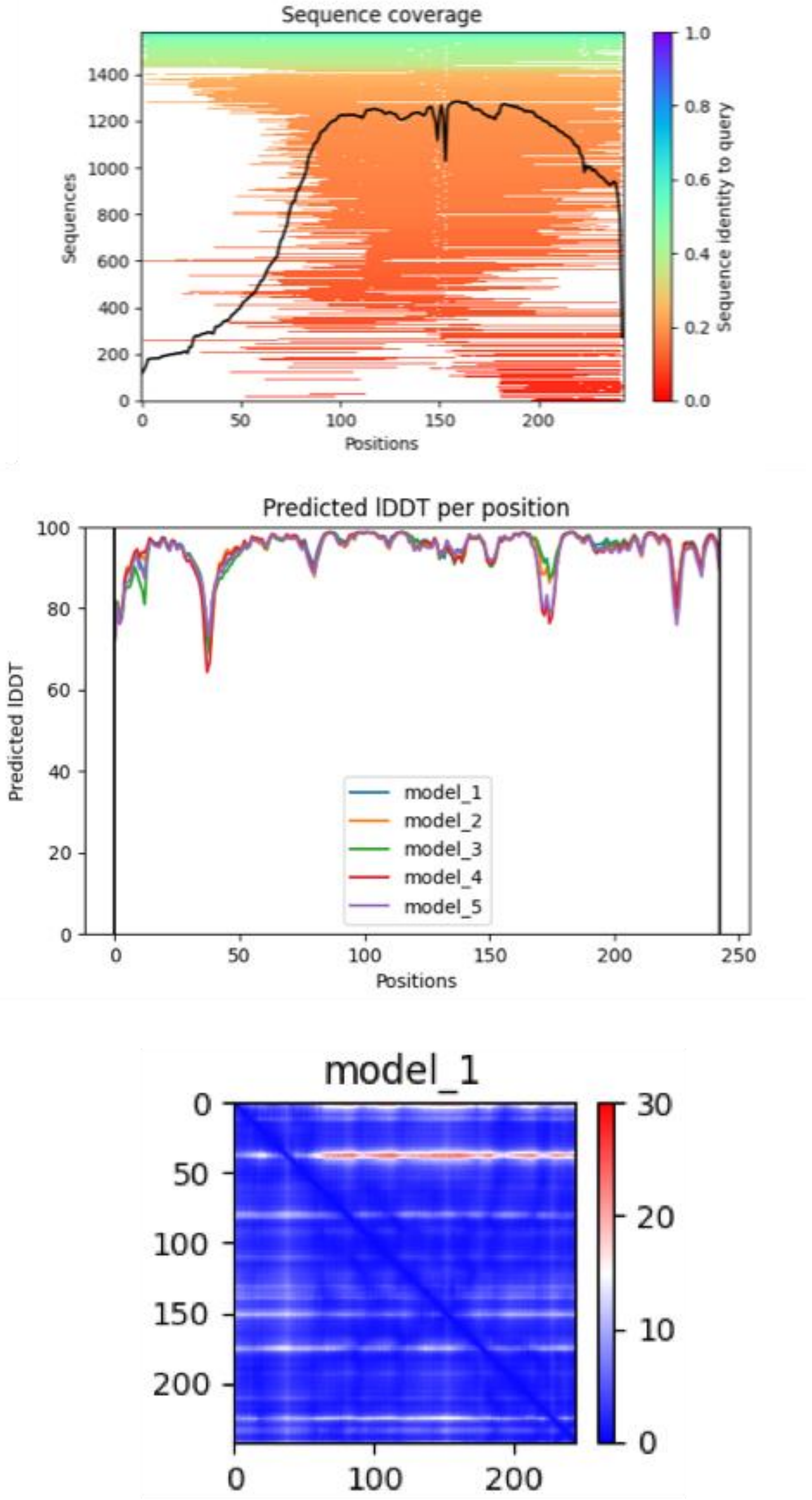

Fig. S7: Output from AlphaFold 2.0 for the CheR$_{like}$ domain model
Five models of the *Td* CheR$_{like}$ domain were generated from the AlphaFold 2.0 Colab notebook (dpmd.ai/alphafold-colab). From the resulting models, Model 1 was chosen for further analysis.

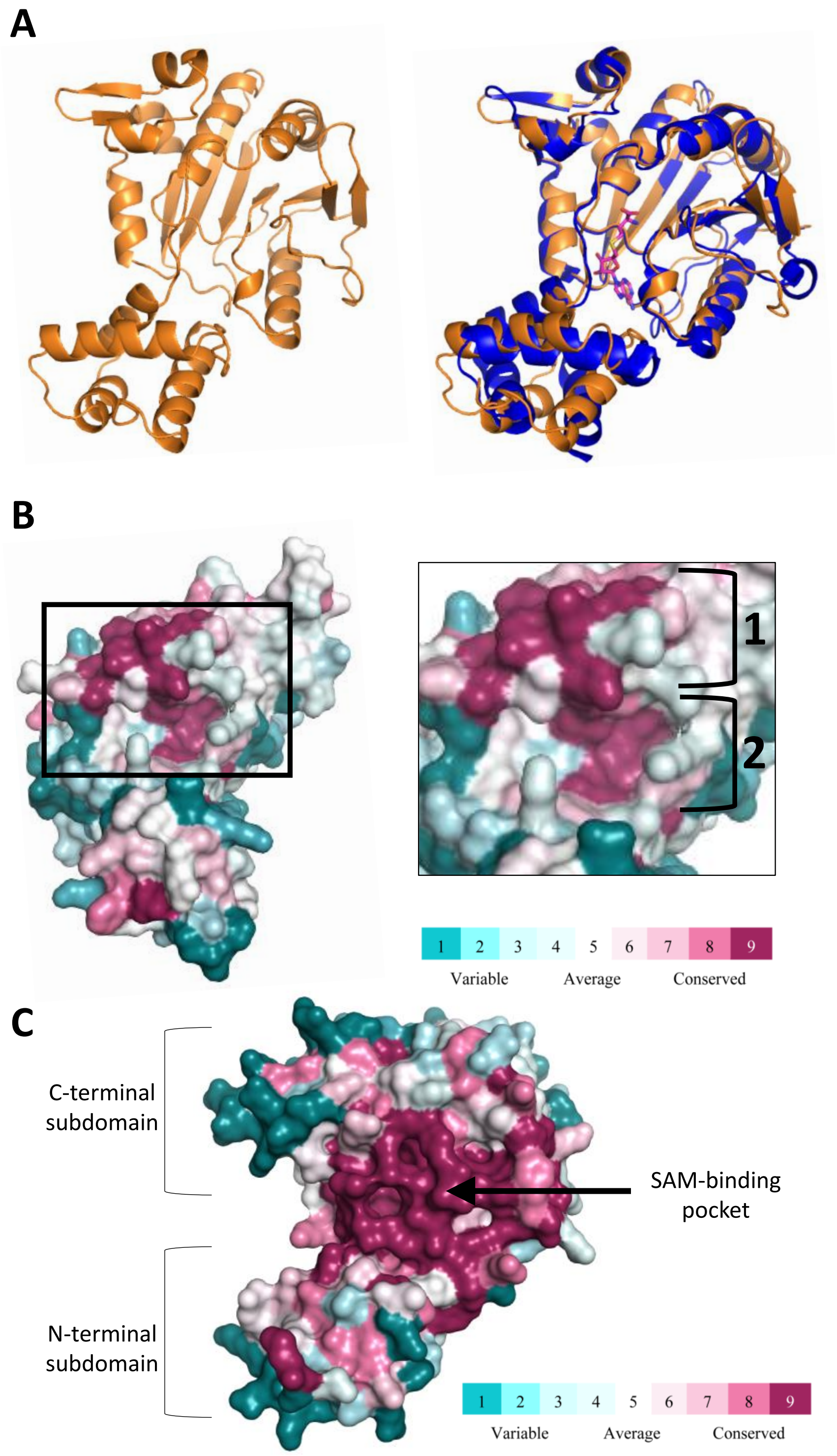

Fig. S8: Structural analysis of the *Td* classical CheR protein model
(A) A model of the *Td* classical CheR protein was generated from the AlphaFold 2.0 Colab notebook (dpmd.ai/alphafold-colab). The model (orange) possesses the same topology and position of the subdomains as previously determined CheR structures (blue, PDBID: 5FTW), which buries the hypothetical SAM pocket. **(B)** Conservation analyses of the model reveals relatively high sequence conservation at the hypothetical SAM-biding pocket and regions known to interact with receptors.

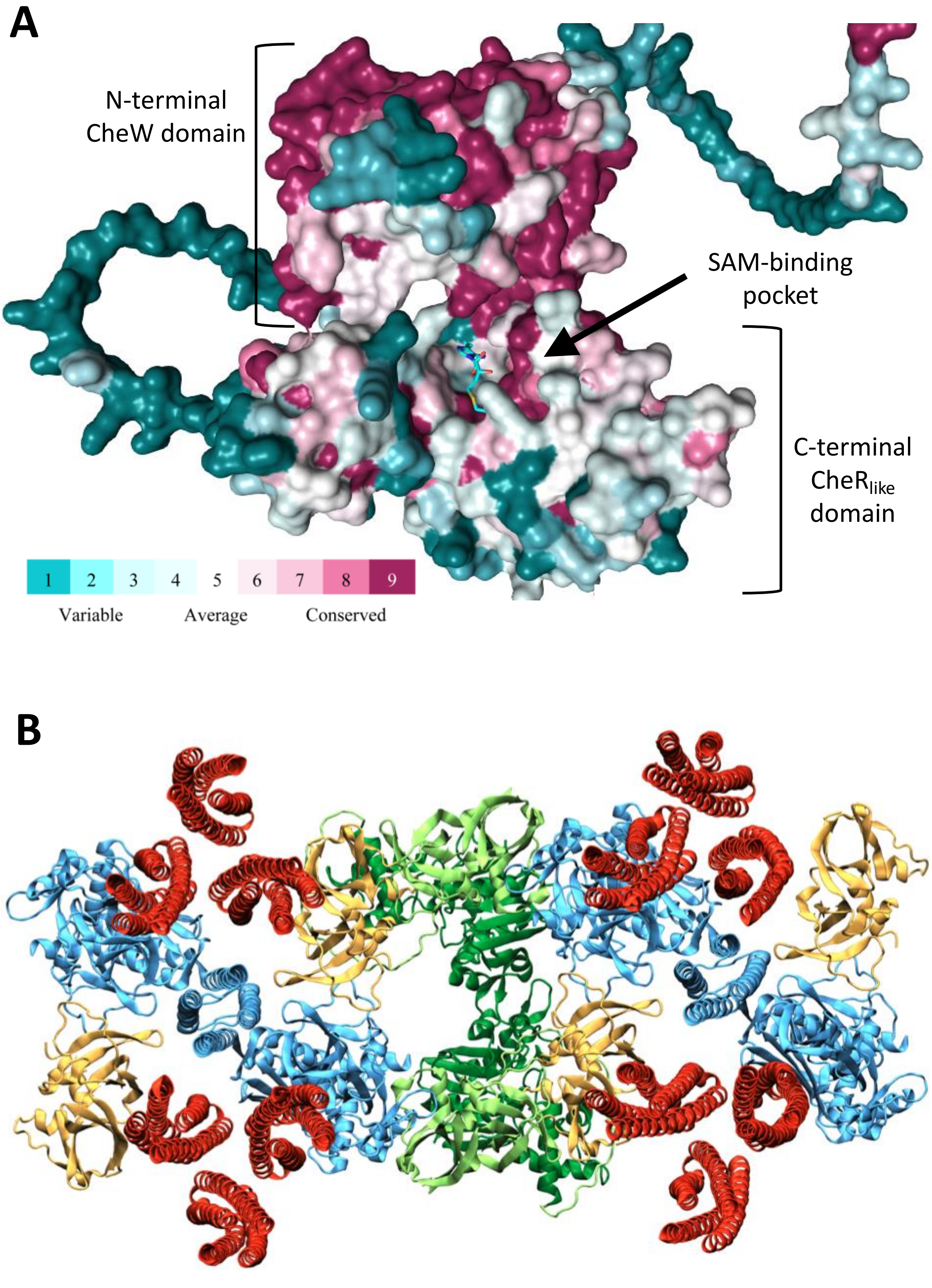

Fig. S9 Structural analyses of CheW
(A) Conservation analysis of CheWS shows that the CheW domain and CheR$_{like}$ domain interact at conserved regions. **(B)** The CheR$_{like}$ domains are predicted to interact with eachother below the hexagonal chemotaxis rings, which places them adjacent to the CheA P4 catalytic domain.

Fig. S10: Protein domain architectures containing CheW domain model without a known function.

The domain architectures are organized by their putative type of biological function. The RR domains are the Pfam Response_reg (PF00072). The architectures are annotated with predicted disordered (gray) and low complexity (blue) regions.

	CheWS	$CheR_{like}$ domain	$CheR_{like}$ domain mut.
SAM Kd (µM)	8.8 +/- 1.1*	21 +/- 4	--
SAH Kd (µM)	17 +/- 3.2	35 +/- 1.1	--

Table S1: Binding assays using SAM and SAH.
Isothermal calorimetry experiments with SAM and SAH show that SAM is the preferred ligand of CheWS. Furthermore, the isolated $CheR_{like}$ domain binds both substrates with lower affinity. Mutation of residues predicted to contribute to ligand binding in the $CheR_{like}$ domain ($CheR_{like}$ domain mut., E297A and D321A) eliminates binding to both SAM and SAH. *This value is from a previous report[29].
